# CD47 blockade augments anti-GD2 driven phagocytosis in vitro but fails to improve in vivo efficacy in immunocompetent, chemoresistant neuroblastoma preclinical models

**DOI:** 10.1371/journal.pone.0353900

**Published:** 2026-10-27

**Authors:** Courtney Himsworth, Thomas Jackson, Chantelle Bowers, Sophie Munnings-Tomes, Gaya Nair, Henrike Muller, Elizabeth Tucker, Amy K. Erbe-Gurel, Paul M. Sondel, Louis Chesler, Robbie Mazjner, John Anderson

**Affiliations:** 1 University College London, Institute of Child Health, London, United Kingdom; 2 Institute of Cancer Research, Sutton, United Kingdom; 3 University of Wisconsin, Wisconsin, United States of America; 4 Dana Farber Cancer Institute, Boston, United States of America; Shanghai Jiaotong University: Shanghai Jiao Tong University, CHINA

## Abstract

CD47 delivers a dominant “Don’t Eat Me” signal that inhibits macrophage‑mediated clearance of tumour cells. Using immunocompetent, chemoresistant neuroblastoma (NB) models, we tested a Fc‑silent CD47 blocker (ALX301) with anti‑GD2 antibody alone and in combination with a clinically aligned temozolomide/irinotecan chemoimmunotherapy backbone. Tumours expressed GD2 and CD47, and bound ALX301. In macrophage coculture assays, anti-GD2 antibody induced dose-dependent phagocytosis, whereas ALX301 or an anti-CD47 antibody alone did not. CD47 blockade in combination with a suboptimal concentration of anti-GD2 antibody showed an additive effect on phagocytosis *in vitro*. *In vivo*, however, ALX301 failed to improve tumour control or survival when added to anti‑GD2 or to chemoimmunotherapy in the dosing regimens and two independent models tested. Toxicity was acceptable, showing only mild, expected red‑cell changes without overt organ injury. This form of CD47 inhibition is therefore mechanistically active *in vitro* but insufficient to enhance anti‑GD2 antibody-based therapy in immunocompetent mice bearing a chemoresistant NB, highlighting the potential need for myeloid‑reprogramming partners.

## Introduction

Neuroblastoma (NB) is the most common extracranial solid tumour of infancy and early childhood, and outcomes for high-risk disease remain poor despite intensive multimodality therapy. Immunotherapy aims to engage host immunity to recognise and eradicate tumour cells. Anti-GD2 monoclonal antibodies, directed against the widely and highly expressed NB antigen disialoganglioside GD2, are now clinically implemented. When paired with chemotherapy such as temozolomide/irinotecan or cyclophosphamide/topotecan, this chemoimmunotherapy approach improves progression-free survival at relapse versus historical cohorts and has become the platform for rational combinations in ongoing trials [1–6].

CD47 is an ubiquitously expressed transmembrane receptor of the immunoglobulin superfamily [7,8]. Its principal ligand, Signal Regulatory Protein alpha (SIRPα), is found on myeloid cells (monocytes, granulocytes, dendritic cells and haematopoietic stem cells) and delivers an inhibitory “self” signal [9]. This function was first established on erythrocytes, for which loss of CD47 marks senescent red cells for clearance by red-pulp macrophages [10]. Across many human cancers CD47 is overexpressed, prompting development of CD47**-**SIRPα blocking agents (antibodies and SIRPα-based binders); comprehensive reviews summarise the preclinical and clinical experience [11–14]. Given the natural expression of CD47 on haemopoietic cells, Fc-competent anti-CD47 agents may also effect haematological toxicity through off-tumour opsonisation. ALX301 was therefore designed as a Fc-silent CD47 blocker, comprising a high affinity SIRPα D1 domain (engineered for increased binding to mouse CD47) fused to mouse IgG1 Fc with the point mutation N297A to abrogate Fcγ receptor engagement while preserving CD47 binding (Fig 1a).

**Fig 1:**
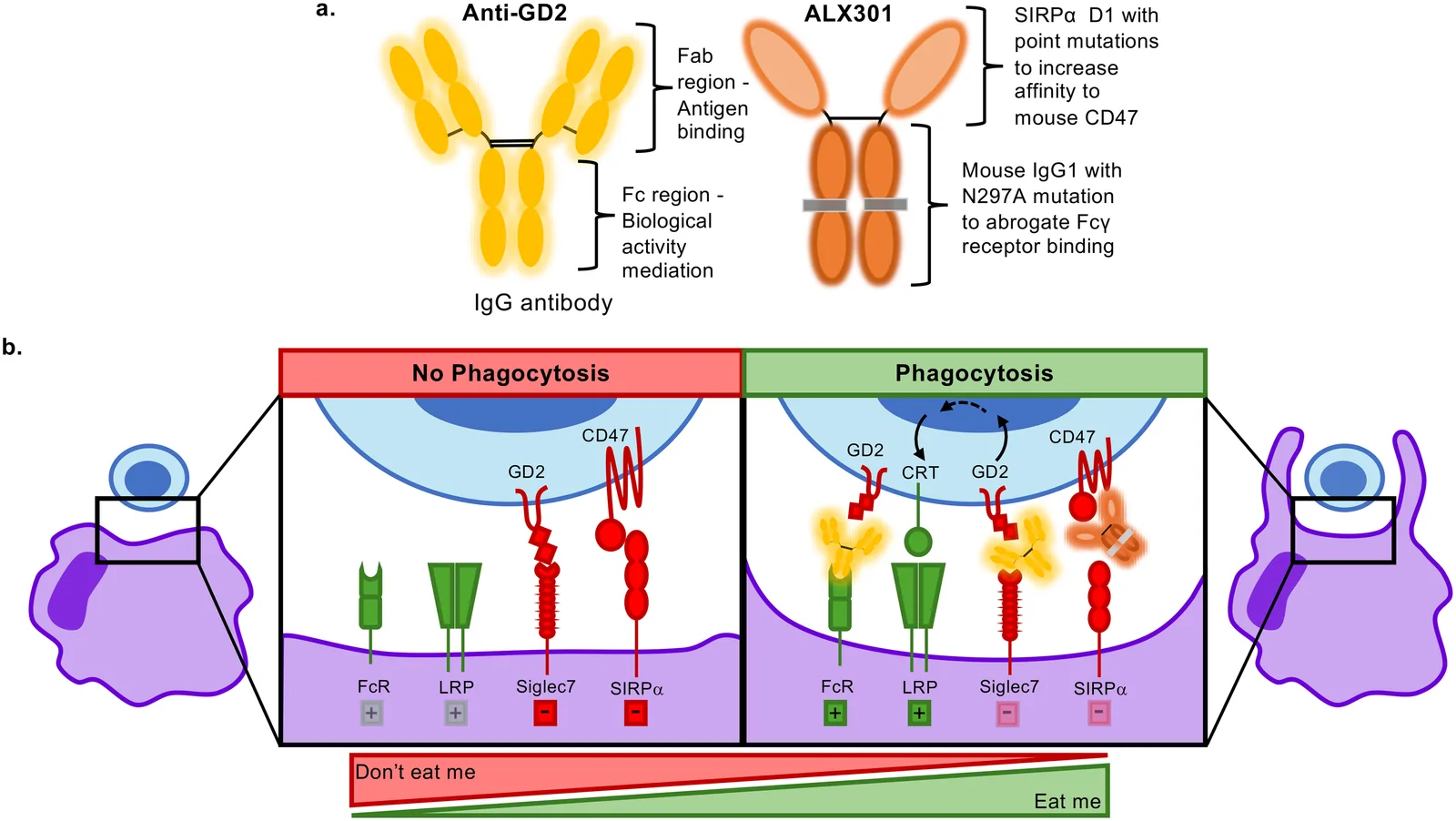
Mechanism of phagocytosis induction by macrophage-targeted immunotherapies. a. Schematic of anti-GD2, a conventional monoclonal antibody, and ALX301, a fusion protein molecule. b. Overview of signalling involved in phagocytosis regulation. Anti-GD2 and ALX301 treatments shift signalling to favour macrophage-mediated tumour cell phagocytosis.

Macrophages clear antibody-opsonised targets through Fcγ receptor-mediated antibody-dependent cellular phagocytosis (ADCP), if pro-phagocytic “Eat-me” cues (e.g., calreticulin) outweigh inhibitory “Don’t Eat Me” signals including CD47. Blocking CD47 can tip this balance toward phagocytosis [15]. At the tumour-macrophage synapse, anti-GD2 provides the opsonising Fc to engage Fcγ receptors. Anti-GD2 also blocks GD2 binding to its inhibitory ligand, Siglec-7, and can increase calreticulin expression. CD47 blockade simultaneously removes the inhibitory brake (Fig 1b). Together, these mechanisms enhance macrophage-mediated tumour clearance, as shown in prior NB studies [16].

Here we validate immunocompetent, chemotherapy-resistant genetically engineered NB mouse models that recapitulate key features of the human NB tumour microenvironment [17]. We use these models to test whether ALX301 augments anti-GD2, and whether any added benefit persists when layered onto a clinically aligned temozolomide/irinotecan chemotherapy backbone. We show that CD47 inhibition increases anti-GD2 driven phagocytosis *in vitro* but these effects are not translated *in vivo***,** failing to improve anti-GD2 monotherapy or chemoimmunotherapy efficacy with regimens tested across two independent models. Both models exhibit a myeloid-rich, suppressive microenvironment, raising the possibility that features of the myeloid niche may contribute to the limited therapeutic benefit observed despite target expression. We therefore suggest that macrophage-reprogramming strategies warrant investigation as a potential means of enhancing CD47-axis therapy in NB.

## Methods

### In vivo establishment of chemoresistant neuroblastoma lines

To model human NB tumours *in vivo*, genetically engineered mouse models (GEMMs) were generated to overexpress MycN and Alk^F1174L^ ligated downstream of the rat tyrosine hydroxylase (*Th*) promoter which developed spontaneous tumours.

The 9464D-GD2 cell line was generated and kindly provided by Paul Sondel group (University of Wisconsin, US). A *Th-MycN*^*+/-*^ GEMM was used to generate the 9464D NB cell line, a model with naturally high resistance to chemotherapy treatment [18]. To more closely model human NB, 9464D cells were modified by viral transduction with GD2/GM2 synthase and GD3 synthase to enable cell-surface GD2 expression, generating the 9464D-GD2 cell line [19]. The GD2/GM2 synthase and GD3 synthase constructs were bicistronic and contained puromycin and blasticidin resistance cassettes, respectively.

Dissociated tumour material used to establish the TAM6 and WIN6 NB cell lines was kindly provided by Louis Chesler’s group at the Institute of Cancer Research, Sutton, UK. These models were derived from spontaneous tumours arising in GEMMs of NB. *Th-MycN* transgenic mice were crossed with *Th-Alk*^*F1174L*^ transgenic mice and the *Th-MycN*^*+/-*^*/Th-Alk*^*F1174L/-*^ offspring exhibited higher tumour penetrance and rapid lethality compared to the *Th-MycN* mice [20].

TAM6 was established from a spontaneous tumour arising in a female *Th-MycN*^+^*^/^*^−^*/Th-Alk*^F1174L/-^ mouse on a 129SvJ/X1 background. To generate a chemotherapy-resistant model, tumours were serially passaged subcutaneously into recipient mice and subjected to escalating cycles of VAC chemotherapy comprising vincristine, doxorubicin and cyclophosphamide [21]. The treatment regimen consisted of four cycles of 0.015 mg/kg vincristine, 0.68 mg/kg doxorubicin and 32 mg/kg cyclophosphamide, followed by two cycles of 0.037 mg/kg vincristine, 1.7 mg/kg doxorubicin and 80 mg/kg cyclophosphamide. Following tumour regrowth after treatment, the tumour was dissociated and established as the TAM6 cell line.

WIN6 was generated from a sixth-generation backcross of *Th-MycN*^+^*^/^*^−^*/Th-Alk^F1174/^*^−^ mice from the 129SvJ/X1 onto the C57BL/6 background. A spontaneous tumour arising in a male mouse was allografted subcutaneously into a fully C57BL/6 recipient and treated intraperitoneally with a single cycle of VAC chemotherapy comprising 0.0015 mg/kg vincristine, 0.68 mg/kg doxorubicin and 32 mg/kg cyclophosphamide. Following tumour regrowth, the tumour was dissociated and established as the WIN6 cell line.

### Cells and culture conditions

The GD2-overexpressing 9464D-GD2 line was maintained under selection with puromycin (6 µg/mL) and blasticidin (7.5 µg/mL) [18,19]. Cells grew adherently in DMEM supplemented with 10% FBS, 1% L-glutamine, 1% non-essential amino acids and 1% sodium pyruvate. Neurosphere lines TAM6 (129SvJ/X1) and WIN6 (C57BL/6) were cultured as suspension spheres in DMEM/F-12 supplemented with 15% FCS, B27 (1X), β-mercaptoethanol (0.05 mM), human EGF (0.01 µg/mL) and human bFGF (0.015 µg/mL). Before *in vitro* or *in vivo* use, live cells were enriched by Ficoll separation.

### Animals

All animals were housed in individually ventilated cages with water and food available *ad libitum* and monitored daily. All procedures were reviewed and approved by a University College London biological services named animal care and welfare officer and named veterinary surgeon, and performed in accordance with UK Home Office project license PP5675666. Research staff involved in animal procedures received appropriate institutional training in mouse handling, welfare monitoring, and recognition of humane endpoint criteria.

### Therapeutics, concentrations and dosing regimens

Temozolomide (TEM), irinotecan (IRI), and anti-GD2 monoclonal antibody (clone 14G2a, Bio X Cell) were used at clinically relevant levels. Human-equivalent doses (HED) and schedules were adapted from phase II studies in relapsed/refractory NB with patient plasma exposures guiding *in vitro* antibody titrations (0–100 nM) [4]. All drugs were administered to mice via intraperitoneal injection throughout. For *in vivo* conversion, human to mouse dose followed Nair *et al.*[22], yielding 100% HED values of 32 mg/kg TEM, 16 mg/kg IRI**,** and 5 mg/kg anti-GD2. We fixed anti-GD2 at 5 mg/kg and titrated TEM/IRI to identify a suboptimal backbone. We evaluated graded HED backbones at 75% (24/12 mg/kg), 50% (16/8 mg/kg), 25% (8/4 mg/kg), and 10% (3.2/1.6 mg/kg) (TEM/IRI values listed as TEM/IRI). To find the anti-GD2 antibody monotherapy window, we titrated anti-GD2 in a four-dose over two-weeks regimen where high was 2.5 mg/kg, medium was 1.25 mg/kg and low was 0.625 mg/kg per dose. Note the high schedule matches the total anti**-**GD2 exposure used with TEM/IRI but distributed over two weeks rather than one. The Fc-silent CD47 blocker ALX301 (ALX Oncology, engineered SIRPα D1 fused to mouse IgG1-N297A) was administered at 30 mg/kg, typically four doses over two weeks, per provider guidance. The commercial anti-CD47 antibody (clone MIAP301, Bio X Cell) was used for *in vitro* assays.

### Isolation of bone marrow derived macrophages (BMDM)

BMDMs were generated *ex vivo* using a method described by Gonzales and Mosser [23]. Briefly, femurs from syngeneic mice were stripped of adherent muscle and connective tissue, cut below the hip joint and above the knee joint then flushed with phosphate-buffered saline (PBS) using a syringe and needle. Red blood cells (RBCs) were lysed using ammonium-chloride-potassium (ACK) lysis buffer for 5 minutes, diluted in PBS then passed through a 70 µm cell strainer to remove debris and 1 x 10^4^ cells were plated on a non-tissue-culture-treated 10 cm^2^ dish with 10 ml DMEM/F-12, 10% FCS, 1X penicillin/streptomycin with macrophage colony-stimulating factor (M-CSF; 20 ng/ml) for 3 days and topped up with a further 5 ml media including M-CSF (20 ng/ml) for a further 4 days. At day 7, BMDMs were harvested by treatment with an enzyme-free cell dissociation buffer (ThermoFisher) and were used in downstream assays.

### *In vitro* phagocytosis assays

Tumour cells were labelled with 2 µM carboxyfluorescein succinimidyl ester (CFSE) for 20 minutes, washed then mixed with BMDMs at 2:1 (tumour:macrophage) in ultra-low-attachment 96-well plates in serum-free medium. Agents were added as: anti-GD2 antibody alone (0–100 nM), CD47 blocker alone (anti-CD47 or ALX301, 0–100 nM), or CD47 blocker (0–100 nM) plus suboptimal anti-GD2 (0.5 nM). After 2 hours incubated at 37 °C 5% CO_2_, phagocytosis was stopped with two 4°C PBS washes and cells were stained with fixable viability dye (near-IR), CD45 (Pacific Blue) and, for macrophage-only controls, F4/80 (AF700). Phagocytosis was quantified by flow cytometry as percentage CFSE⁺CD45 ⁺ macrophages and normalised by subtracting the background phagocytosis measured in the control condition in absence of any drug (0nM).

### *In vivo* studies and treatment combinations

For each treatment arm, n (number of mice) is reported in the figure panels/legends. No formal a priori power calculation was performed because effect sizes in these models were unknown. Group sizes (n = 4–8/arm) were chosen from prior variability and 3Rs principles to detect large, practice-changing effects; key null findings were replicated in independent cohorts and in both models. Mice of 6–12 weeks old were tumour engrafted. To ensure a uniform tumour-bearing cohort at treatment start, an additional ~40% of mice were engrafted; animals outside prespecified tumour-size limits were not enrolled. All treated animals enrolled in the studies were included in the final analysis.

TAM6 was derived from a tumour in a female mouse and WIN6 from a male mouse; therefore all subsequent allografts were sex-matched within their respective strains to avoid confounding immune responses to sex-linked (e.g., H-Y) antigens.

For subcutaneous engraftment, mice were briefly anaesthetised with gaseous isoflurane for approximately 5 minutes to allow flank shaving for early tumour detection and measurement, ear notching for identification, and tumour cell implantation. Animals were closely monitored until full recovery from anaesthesia. TAM6, WIN6, or 9464D-GD2 cells were injected into the right flank in 50% Matrigel and 50% PBS at optimised doses: 2x10^5^ cells/mouse for TAM6 and 2x10^6^ cells/mouse for 9464D-GD2 (as reported previously [19,21]) and 5x10^5^ cells/mouse for WIN6 yielded consistent growth in C57BL/6J mice. Tumour volume was calculated as (length x width^2^)/2. Mice were enrolled at ~10–80 mm³ (unambiguous by calliper measurement). Animals were ranked by baseline volume, binned from smallest to largest, and randomly assigned to groups via a list generator to balance means/SDs across arms.

Temozolomide and irinotecan stocks were stored at −20 °C in DMSO, thawed on ice, diluted in PBS immediately prior to dosing, and co-administered intraperitoneally as a single injection. Anti-GD2 and ALX301 were prepared in PBS, stored at 4 °C at working concentration, and were delivered intraperitoneally; when co-dosed, they were mixed immediately prior to and given as a single injection. Each experiment included a matched vehicle-control group: mice received injections on the same schedule as the most intensive regimen, with the appropriate vehicle (PBS or DMSO/PBS) substituted for drug.

### Blood collection and histology

Peripheral blood was collected from the saphenous vein into EDTA tubes and submitted to IDEXX UK for complete blood count (CBC) analysis. CBCs were obtained at the end of treatment on day 19 or at the humane endpoint for animals with rapidly growing tumours requiring euthanasia prior to the day 19 end of treatment-scheduled blood sampling.

For histological assessment, heart, lung, kidney, liver, spleen and tumour tissues were harvested following humane euthanasia and fixed in 4% paraformaldehyde for approximately 24 hours. Tissues were routinely processed and paraffin-embedded as a paid service provided by Great Ormond Street Hospital, after which sectioning and haematoxylin and eosin (H&E) staining were performed. Three tissue sections from each organ were mounted per slide. Entire slides were digitised using a NanoZoomer S60 digital slide scanner (Hamamatsu Photonics), and whole tissue sections were qualitatively examined for overt morphological abnormalities and evidence of treatment-associated tissue damage. No formal histopathological scoring was performed, and sections were not independently reviewed by a veterinary pathologist. Where histological images are presented in figures, representative regions from the scanned whole-slide images are shown.

### Humane endpoints and monitoring

Humane endpoints were predefined and applied to minimise suffering. Animals were observed daily, with additional monitoring during treatment where required, and any animal identified as nearing or meeting endpoint criteria was euthanised immediately upon detection, with no delay between endpoint recognition and euthanasia. Humane endpoints included any single tumour dimension >15 mm, ulceration at the engraftment site, or treatment-related toxicity, defined as >15% body-weight loss or a moderate grimace score. After tumour engraftment, body weight and tumour dimensions were recorded twice weekly using callipers, and animals were monitored closely during treatment.

Mice were euthanised at humane endpoint or study completion by CO_2_ asphyxiation or cervical dislocation. Death was confirmed by cessation of circulation following severance of the femoral artery. No mice died before reaching endpoint criteria. For all euthanised animals, the reason for euthanasia was tumour burden rather than treatment-related toxicity in the absence of tumour progression.

The primary outcome, had a power calculation been performed, was overall survival, defined as time from treatment start to euthanasia at protocol-defined humane endpoint due to tumour outgrowth. Survival curves therefore depict protocol-defined overall survival, as indicated in each experimental figure. Secondary outcomes included body-weight change and, where performed, CBCs and organ histology. Assessments for euthanasia and calliper measurements were performed by investigators blinded to treatment allocation.

### Tumour processing

Tumours were dissected from euthanised mice and immediately transferred into ice-cold PBS. Tissue was minced into small fragments using a scalpel and dissociated in Accumax cell detachment solution (Sigma-Aldrich) for 10 minutes at room temperature with gentle agitation on a roller. Following digestion, samples were minced through a 70 µm cell strainer with the back of a syringe plunger to generate a single-cell suspension. Pelleted cells were RBC-lysed using ACK lysis buffer for 5 minutes at room temperature. Cells were then washed in PBS, pelleted by centrifugation and were prepared for analyses by flow cytometry.

### Flow cytometry

All flow cytometry staining was performed in PBS. Fixable viability dye and antibodies were added together and incubated for 20 minutes at 4 °C. Data were analysed using FlowJo version 10.10.1. Flow cytometry data were sequentially gated to exclude debris and doublets and to identify viable cells before application of the relevant lineage- and population-specific gates. Debris was excluded based on forward- and side-scatter characteristics (FSC/SSC). Singlet populations were identified by forming the expected diagonal population on FSC-based height/area plots. Viable cells were defined as those negative for the fixable viability dye. Representative examples of this gating strategy are shown in S1 Fig.

Flow cytometry panels were designed to minimise spectral overlap between fluorochromes. Where compensation was required, UltraComp eBeads Plus Compensation Beads (Thermo Fisher Scientific) were stained with 1 µl of the relevant antibody and acquired alongside experimental samples. Compensation matrices were calculated on the flow cytometer. Fluorescence-minus-one controls were used to establish gating boundaries.

Where tumour-cell surface expression of therapeutic target antigens was assessed, cells were stained with anti-mouse anti-GD2 (14G2a; BioLegend), anti-mouse anti-CD47 (MIAP301; BioLegend) or ALX301 for 45 minutes followed by anti-mouse anti-IgG1 (RMG1−1; BioLegend), as appropriate. Corresponding isotype-control antibodies were obtained from BioLegend and used to define background staining. Samples were acquired on a CytoFLEX flow cytometer (Beckman Coulter) on the same day as staining.

Single-cell tumour suspensions were treated with Fc block for 10 minutes, followed by staining with a fixable viability dye (Thermo Fisher) and the relevant surface-antibody master mix (detailed in Table 1) for 20 minutes at 4 °C. Samples were then fixed with 4% paraformaldehyde for 10 minutes at 4 °C and washed with PBS. For intracellular staining, fixed cells were processed using BD Biosciences Perm/Wash Buffer according to the manufacturer’s instructions. Cells were washed twice with 1X permeabilisation buffer, incubated with the relevant intracellular antibodies for 20 minutes at 4 °C, then washed a further two times with 1X permeabilisation buffer. Samples were acquired on a BD FACSymphony A5 within 3 days of fixation.

**Table 1:** Antibodies used across all TME panels.

*Panel*	*Marker*	*Fluorochrome*	*Clone*	*Manufacturer*
*Overview*	*Live/Dead*	*Blue*		*ThermoFisher*
	*CD19*	*BUV563*	*1D3*	*BD Horizon*
	*NKp46*	*BV421*	*29A1.4*	*BioLegend*
	*CD11b*	*BV605*	*M1/70*	*BioLegend*
	*CD45*	*FITC*	*30-F11*	*BioLegend*
	*CD3*	*PE/Fire700*	*17A2*	*BioLegend*
	*GD2*	*APC*	*14G2a*	*BioLegend*
*Myeloid*	*Live/Dead*	*Blue*		*ThermoFisher*
	*CD11b*	*BV605*	*M1/70*	*BioLegend*
	*F4/80*	*BV711*	*BM8*	*BioLegend*
	*CD45*	*FITC*	*30-F11*	*BioLegend*
	*CD80*	*PE*	*16-10A1*	*BioLegend*
	*Ly6G*	*PE/Dazzle594*	*1A8*	*BioLegend*
	*CD206*	*APC*	*C068C2*	*BioLegend*
	*Ly6C*	*APC/Cy7*	*HK1.4*	*BioLegend*

### Statistical analysis

Data was processed in Microsoft Excel and graphed in GraphPad Prism. Each point represents a biological replicate**.** Bars show mean ± s. d. unless otherwise indicated. Group comparisons used two-way ANOVA with Tukey’s multiple comparisons correction; exact P values are shown for significant contrasts. Survival analyses (where applicable) used log-rank (Mantel-Cox) tests. Hazard ratios were estimated using the Mantel-Haenszel method and are reported with 95% confidence intervals within figure legends.

## Results

### Establishment and immune contexture of chemoresistant, immunocompetent NB models

NB is an embryonal tumour derived from neural crest progenitor cells. *MycN* gene amplification and *Alk* activating mutations are associated in promoting aggressive tumours in ultra-high-risk NB patients [24,25]. To model these tumours *in vivo*, GEMMs have been generated to overexpress MycN and Alk^F1174L^ downstream of the rat *Th* promoter [20,26]. Genes downstream of the *Th* promoter are expressed in committed sympathetic precursor cells of the neural crest. Expression of oncogenes *Th*-*MycN* and *Th-Alk*^*F1174L*^ give rise to tumours in the sympathetic ganglia and adrenal gland, locations commonly seen in human NB. We generated chemoresistant NB cell lines from *Th-MycN/Th-Alk*^*F1174L*^ spontaneous tumours by sequential addition of vincristine, doxorubicin and cyclophosphamide to establish TAM6 cells on 129SvJ background (previously described [21]), and a new model, WIN6, on C57BL/6 background for syngeneic use. A *Th-MycN* derived 9464D line on C57BL/6 background was transduced to overexpress GD2 (9464D-GD2) for selected comparisons [19] (Fig 2a).

**Fig 2:**
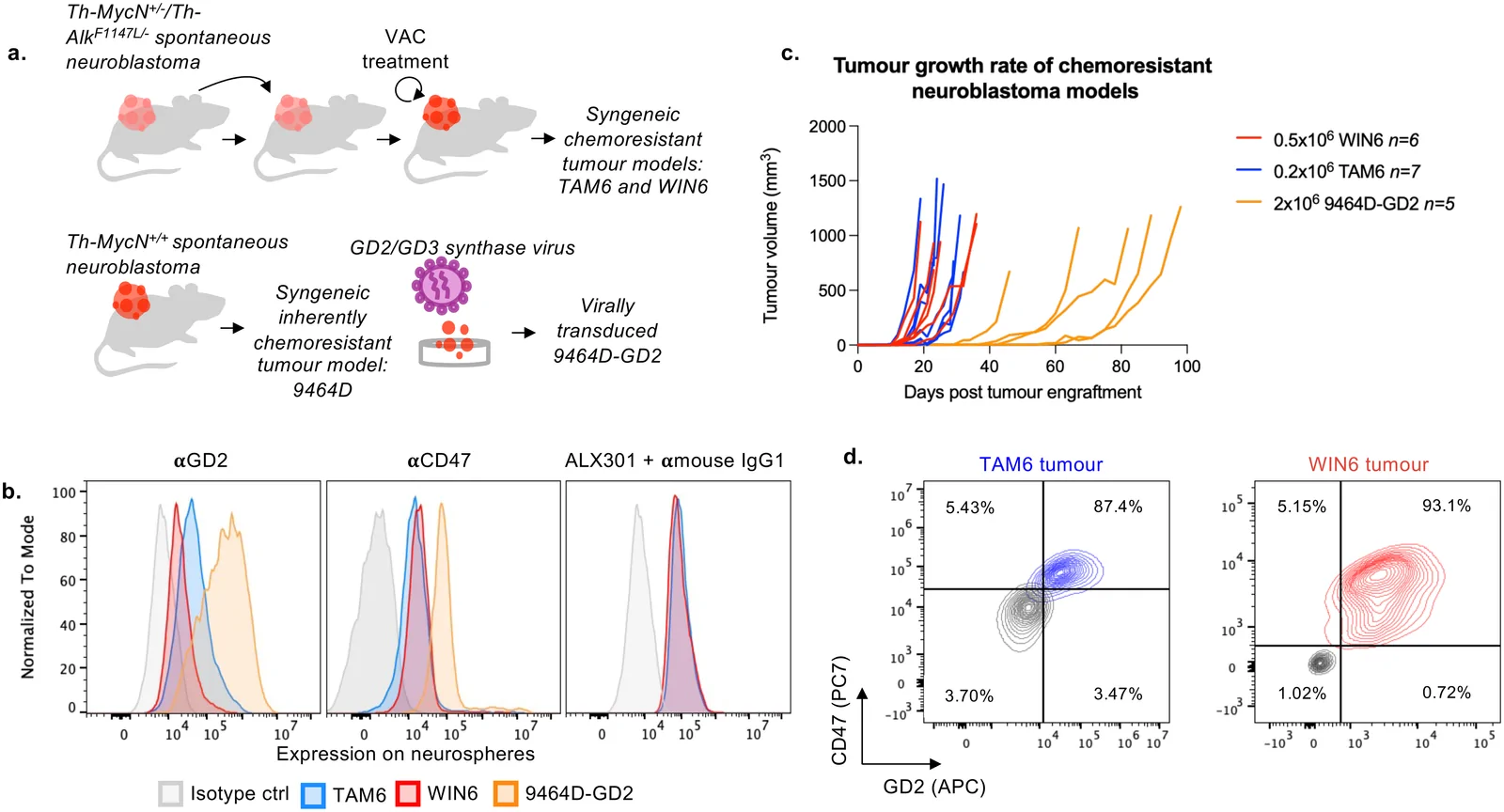
Establishment and characterisation of immunocompetent, chemoresistant neuroblastoma models. **a.** *Schematic of chemoresistant neuroblastoma cell line generation; TAM6, WIN6 and 9464D-GD2.* **b.** *Flow cytometry confirms target antigen expression on TAM6, WIN6 and 9464D-GD2 cells, showing surface GD2 and CD47. ALX301 binding to tumour cells was detected using secondary anti-mouse IgG1 staining.* **c.** *Tumour growth kinetics of subcutaneously engrafted TAM6, WIN6 and 9464D-GD2 cells.* **d.** *Flow cytometric contour plots to demonstrate freshly dissected and stained TAM6 and WIN6 tumours also express GD2 and CD47*.

By flow cytometry, TAM6 and WIN6 expressed GD2 and CD47, and ALX301 bound tumour cells (Fig 2b). Optimised subcutaneous engraftment yielded reproducible growth for all models although 9464D-GD2 grew slower than TAM6 or WIN6 (Fig 2c). Dissected and freshly stained TAM6 and WIN6 tumours confirmed *in vivo* GD2 and CD47 expression (Fig 2d).

To compare immune composition across tumour models, t-distributed stochastic neighbour embedding (t-SNE) analysis was performed on concatenated flow cytometry data from TAM6 and WIN6 tumours. This analysis provided a global visual representation of the TME and revealed distinct immune composition between tumour models. The most striking visual difference emerging from the t-SNE plots was the enhanced myeloid infiltration and decreased T cell population in TAM6 tumours, when compared to WIN6 (Fig 3a). Within the myeloid compartment, both models contain immunosuppressive populations, including MDSCs and M2-like macrophages. Quantitative analysis confirmed these differences and demonstrated that TAM6 tumours were significantly enriched for myeloid populations, whereas WIN6 tumours contained relatively higher proportions of T cells (Fig 3b; gating in S1 Fig).

**Fig 3:**
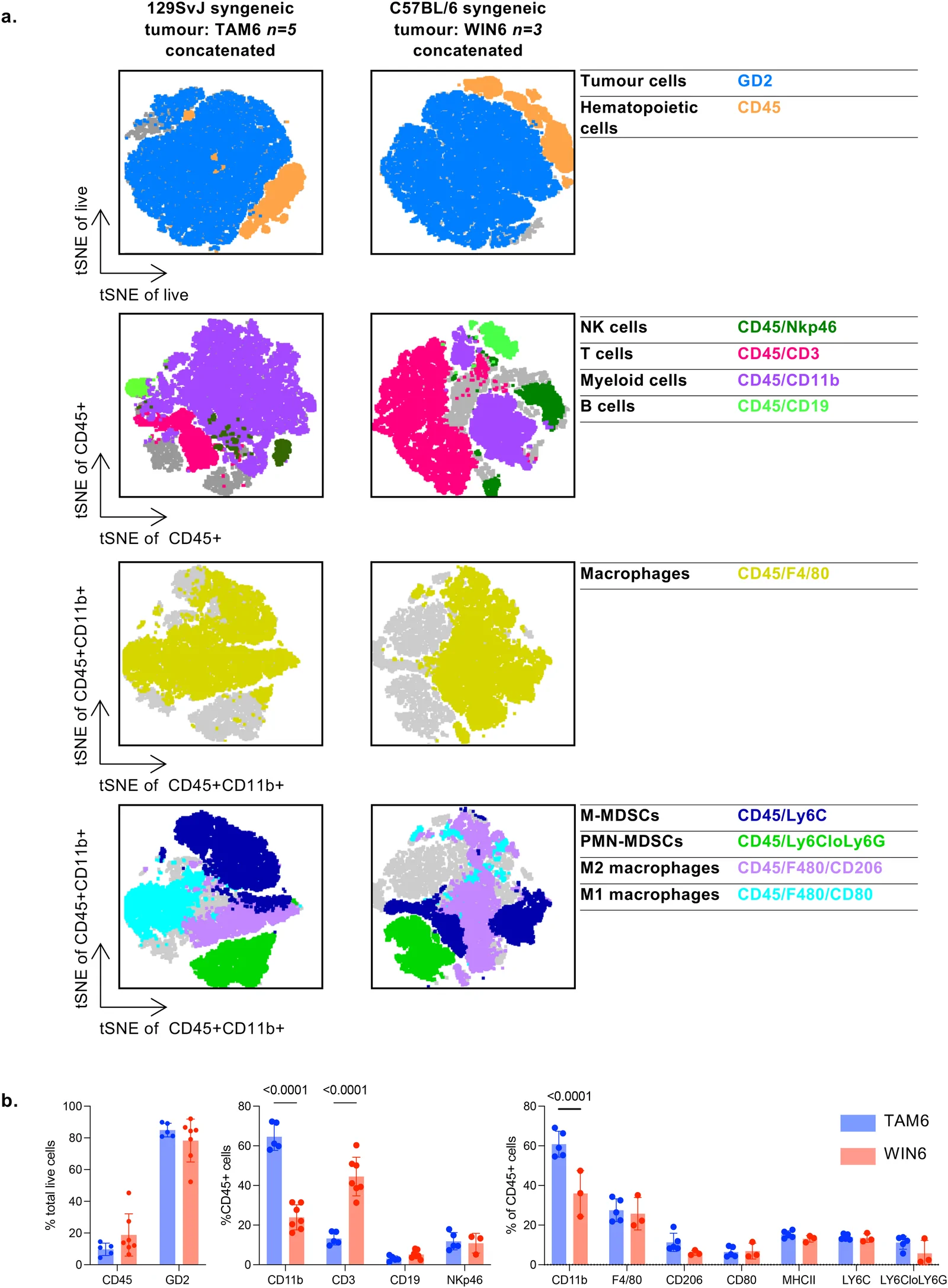
Immune profiling of TAM6 and WIN6 tumours. **a.** *Immune profiling of TAM6 and WIN6 tumours by high-parameter flow cytometry and dimensionality reduction by t-Distributed Stochastic Neighbour Embedding (t-SNE) analysis.* **b.** *TAM6 tumours displayed a significantly larger myeloid compartment within the CD45 gated cells, compared with WIN6*.

### CD47 blockade augments anti-GD2 mediated phagocytosis *in vitro*

To test whether CD47 blockade augments anti-GD2 antibody induced ADCP in these chemoresistant models, CFSE-labelled tumour cells were co-cultured *in vitro* with syngeneic bone marrow derived macrophages at 2:1 tumour:macrophage ratio for 2 hours. Anti**-**GD2 alone induced dose-dependent phagocytosis in both TAM6 and WIN6 models, whereas ALX301 or anti-CD47 (MIAP301) alone did not increase uptake above baseline. Combining either form of CD47 blockade with suboptimal anti-GD2 (0.5 nM) produced a clear additive increase in phagocytosis of TAM6 and WIN6 across biological replicates (Fig 4a, 4b). Based on the percentage uptake of CFSE, the TAM6 model is approximately 5-fold less susceptible to phagocytosis than the WIN6 model (Fig 4b). By contrast, 9464D-GD2, despite high GD2 and CD47 cell surface expression, was resistant to anti-GD2 ± CD47 blockade in the same assay, even when a 10-fold greater concentration of anti-GD2 was used, despite its being susceptible to antibody-dependent cell cytotoxicity (ADCC) by peripheral blood mononuclear effector cells in prior work [19] (Fig 4c, 4d). We tested whether conditioned medium from 9464D-GD2 cultures inhibited anti-GD2 ADCP of WIN6 cells. It did not, indicating that soluble inhibitors released by these 9464D-GD2 cells were not the mechanism behind the resistance of this tumour to ADCP in these *in vitro* assays (Fig 4e).

**Fig 4:**
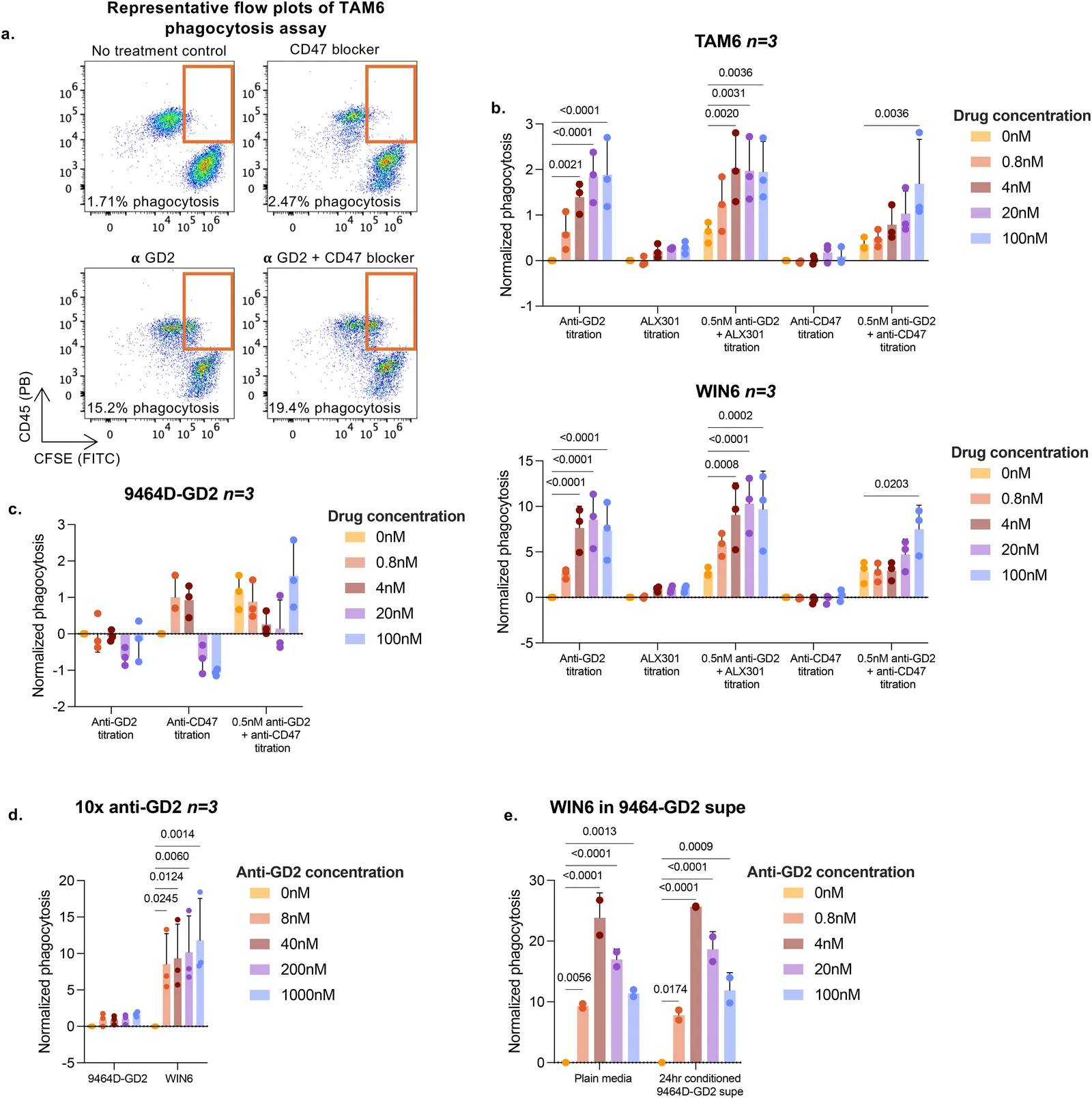
CD47 blockade enhances anti-GD2 mediated phagocytosis in vitro of TAM6 and WIN6 tumour lines. **a.** *Representative flow cytometry plots of phagocytosis assays. Two populations can be seen, tumour cells (CFSE population) and syngeneic bone marrow–derived macrophages (CD45 population). Macrophage uptake of tumour cells was quantified as CFSE*^+^*CD45*^+^ *events indicated by the orange box***. b.** *Quantification of phagocytosis across independent biological replicates (n*=*3 per model). Suboptimal anti-GD2 antibody (0.5 nM) was combined with CD47 blockade using ALX301 or the commercial anti-CD47 antibody.* **c.** *In vitro phagocytosis assays with 9464D-GD2 tumour cells and syngeneic macrophages.* **d.** *Increasing anti-GD2 concentration tenfold did not overcome resistance in 9464D-GD2 cells.* **e.** *Phagocytosis of WIN6 tumour cells was unaffected by conditioned supernatant from 9464D-GD2.*

*Error bars represent mean ± SD; statistical significance between three sources of macrophages was determined using two-way ANOVA with Tukey’s multiple comparison test*.

### CD47 blockade does not improve anti-GD2 antibody or chemoimmunotherapy *in vivo*

As CD47 blockade augmented anti-GD2-induced ADCP *in vitro* for TAM6 and WIN6 tumour cells, we next asked whether this would translate to anti-tumour effects *in vivo.* We first modelled the clinically used chemoimmunotherapy schedule by dosing TEM/IRI on consecutive days 1–5 with anti-GD2 on days 1 and 5. Dose titration of TEM/IRI identified a significant survival advantage with 10% of the HED, TEM/IRI (3.2 mg/kg / 1.6 mg/kg) with anti-GD2 (5 mg/kg) in the WIN6 model, which produced delayed growth without cures (Fig 5a). All higher doses produced widespread complete responses (CR) and so 10% HED was used for subsequent combination studies as a partially efficacious dosing regimen. Due to its proposed superior toxicity profile compared with anti-CD47 antibodies, we selected ALX301 as a CD47 targeting agent. Adding ALX301 (30 mg/kg) to this chemoimmunotherapy backbone did not improve tumour control or survival in either WIN6 or TAM6 models (Fig 5b).

**Fig 5:**
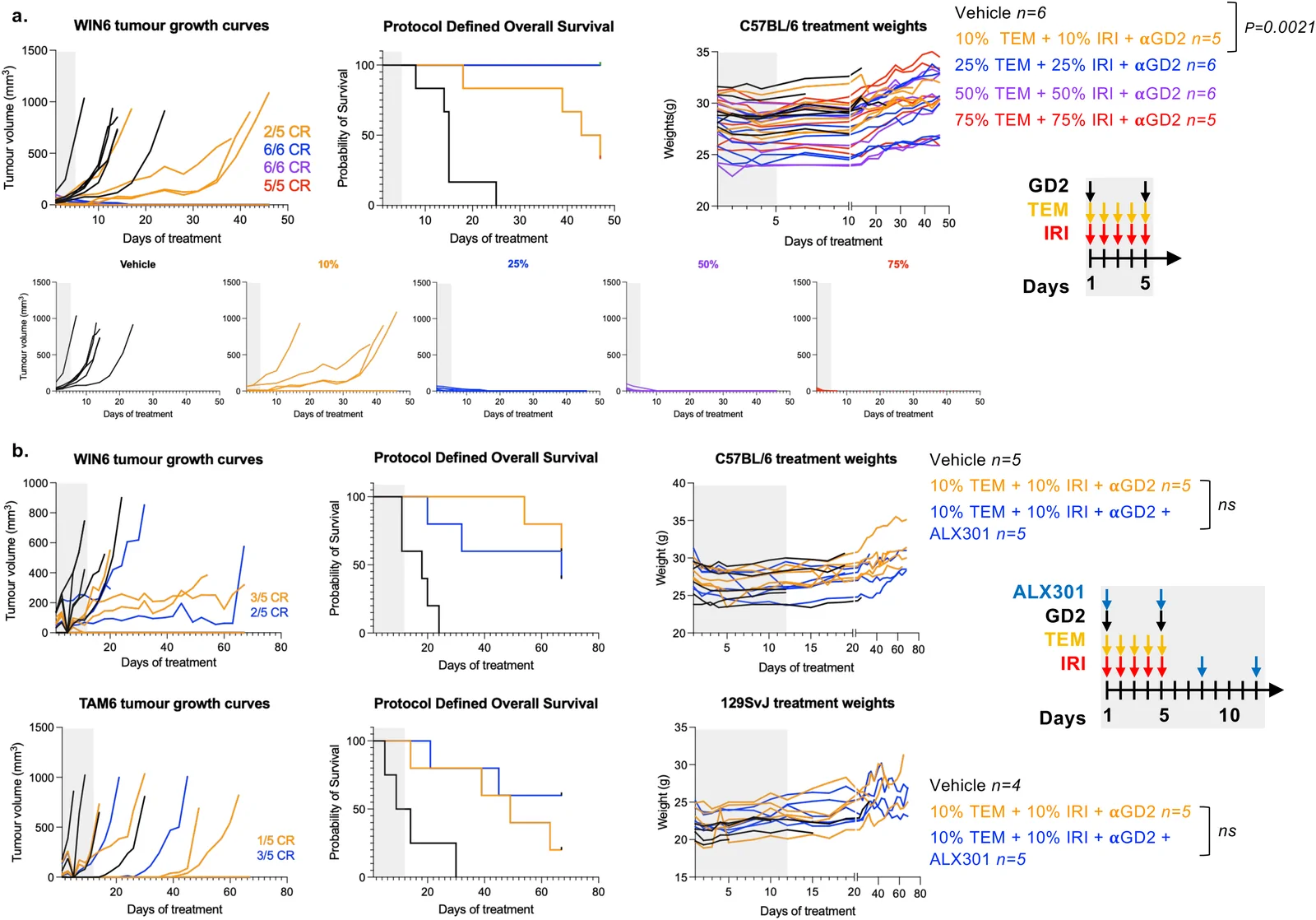
Initial analyses of ALX301 in combination with partially effective chemoimmunotherapy. **a.** *WIN6 tumour growth curves, survival, and treatment group weights following dose titration of temozolomide (TEM) plus irinotecan (IRI) in combination with anti-GD2 (5 mg/kg). Percentages shown are that of HED for TEM and IRI. The log-rank P value is shown for survival comparison between vehicle and 10% HED groups.* **b.** *Tumour growth curves, survival, and treatment group weights are shown for combination of ALX301 (30 mg/kg) with the partially efficacious chemoimmunotherapy regimen in both WIN6 and TAM6 models. For the prespecified survival comparison of chemoimmunotherapy backbone versus backbone* + *ALX301, the hazard ratio was 2.06 (95% CI 0.33-12.92; log-rank P* = *0.4405) for WIN6 and 0.44 (95% CI 0.09-2.19; log-rank P* = *0.3135) for TAM6.* Treatment schemas are shown on the right.

Recognizing that ALX301 was not designed for monotherapy use, we hypothesized that a regimen with prolonged anti-GD2 treatment to allow ALX301 and anti-GD2 co-administration might be necessary to achieve the synergistic effect required for macrophage activation and effective tumour cell clearance. An anti-GD2 monotherapy (1.25 mg/kg) dosed four times over two weeks was partially efficacious in both WIN6 and TAM6 models (S2 Fig). However, when co-administered with ALX301 (30 mg/kg), no additional efficacy was observed compared with anti-GD2 alone in either model (Fig 6a, 6b). In WIN6, the anti-GD2-only group showed a significant survival advantage over the combination group, including one complete response; however, this finding was not reproduced in TAM6 or in subsequent experiments, and therefore does not provide consistent evidence that ALX301 antagonises anti-GD2 activity.

**Fig 6:**
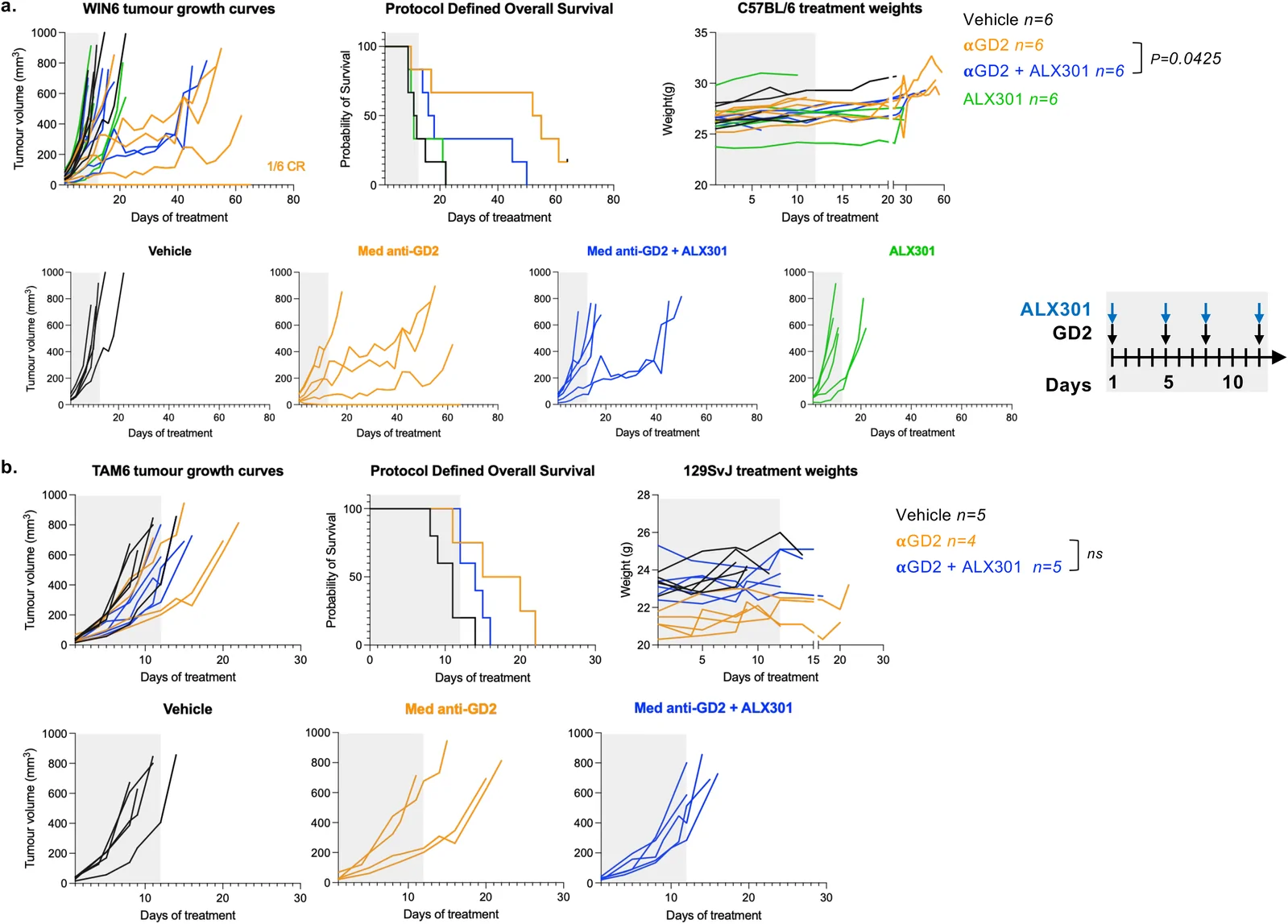
CD47 blockade does not enhance the efficacy of suboptimal anti-GD2 monotherapy *in vivo.* **a.** *WIN6 tumour growth curves, survival, and treatment group weights following treatment with vehicle, suboptimal anti-GD2 (1.25 mg/kg), ALX301 (30 mg/kg), or the combination of anti-GD2 plus ALX301. Anti-GD2 suboptimal dose finding results are shown in supplementary figure* 2*. For the prespecified survival comparison of anti-GD2 versus anti-GD2* + *ALX301, the hazard ratio was 4.43 (95% CI 1.52-18.68; log-rank P* = *0.0425).* **b.** *Equivalent experiment in TAM6 tumours, with treatment groups as indicated. For the prespecified survival comparison of anti-GD2 versus anti-GD2* + *ALX301, the hazard ratio was 2.877 (95% CI 0.57-14.36; log-rank P* = *0.1996)*. Treatment schedule is shown on the right.

### Intensified CD47 blockade with chemoimmunotherapy showed favourable safety profile but no additional benefit

To investigate whether increased exposure could unmask synergy, we extended treatment with an additional two doses of anti-GD2 and ALX301 layered onto the TEM/IRI backbone in the WIN6 model. Efficacy remained unchanged with ALX301 addition (Fig 7a). The complete response rate was numerically lower in the chemoimmunotherapy plus ALX301 group than in the backbone group (4/7 versus 5/7, respectively); however, this difference was not accompanied by a significant survival difference. CBCs revealed small but significant decreases in haemoglobin and haematocrit (RBC %) with CD47 blockade albeit with limited sampling timepoints, consistent with on-target RBC effects, without effects on clinical condition, grimace scores, or body weight (weights summarised as mean ± SD in Fig 7a) (Fig 7b). Histopathology of heart, lung, kidney, liver, spleen, and tumour showed no immediate tissue injury (Fig 7c). Together, these findings suggest that while this regimen of ALX301 does not appear to introduce systemic toxicity when combined with the chemoimmunotherapy backbone, within these two immunocompetent neuroblastoma models, it also does not enhance the efficacy of the treatment compared to the chemoimmunotherapy backbone alone.

**Fig 7:**
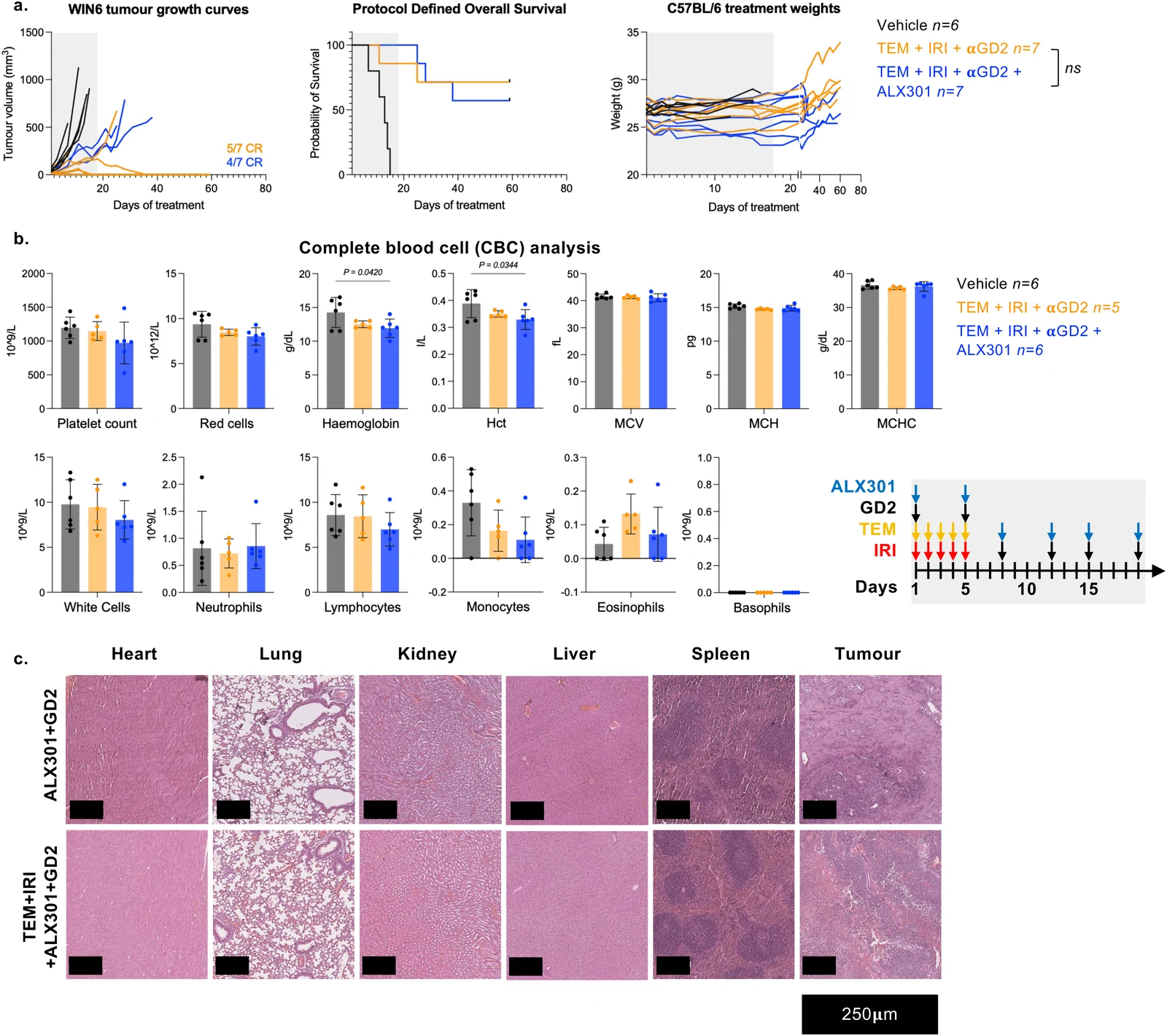
Impact of chemotherapy addition to anti-GD2 and ALX301 on toxicity and efficacy. **a.** WIN*6 tumour growth curves, survival, and treatment group weights following an extended anti-*GD*2 and* ALX*301 treatment schedule combined with chemotherapy (as in* Fig 5*). For the prespecified survival comparison of chemoimmunotherapy backbone versus backbone* + ALX*301, the hazard ratio was 1.37 (95%* CI *0.23-8.16; log-rank P* = *0.7307).* **b.** *To assess toxicity, peripheral blood samples were collected from treated mice either prior to tumour-related death or at the end of treatment, and complete blood counts (CBCs) were performed. Each point represents an individual mouse. Error bars represent mean ± SD; statistical significance was determined using one-way ANOVA with Tukey’s multiple comparison test*. **c.** *Histological analysis of haematoxylin and eosin (H&E) stained sections from heart, lung, kidney, liver, spleen, and tumours. Representative images are shown at 5x magnification, with scale bar representing 250 µm.*

## Discussion

We provide a translational stress test of CD47 blockade in combination with anti-GD2 immunotherapy or chemoimmunotherapy in immunocompetent, chemoresistant NB mouse models. A key contribution of this study is the introduction and immune mapping of two immunocompetent, chemoresistant neuroblastoma models, WIN6 (C57BL/6) and TAM6 (129SvJ). Both models express the clinical targets GD2 and the inhibitory ligand CD47, engraft reproducibly, and in untreated tumours analysed outside the therapeutic studies, contain substantial myeloid populations, including MDSCs and M2-like macrophages, with TAM6 displaying a particularly myeloid-dominant immune composition. These features are consistent with aspects of the immune landscape described in human neuroblastoma and make WIN6 and TAM6 useful platforms for investigating tumour-immune interactions in an immunocompetent setting. These features make WIN6 and TAM6 useful beyond the CD47 question: they enable clinically aligned dosing studies and testing of new combinations, and their intact TME allows interrogation of cell-cell interactions by flow, spatial, and single-cell readouts. The observation that CD47 blockade did not enhance anti-GD2 antibody or chemoimmunotherapy in these stringent models is informative. Together, WIN6 and TAM6 provide a rigorous, disease-relevant platform to derisk combinations, define mechanisms of resistance, and support translational applicability.

Using macrophages derived from bone marrow of healthy mice, an Fc-silent CD47 inhibitor (ALX301) enhanced anti-GD2 driven phagocytosis *in vitro*, confirming the capacity of CD47 blockade to augment macrophage-mediated tumour-cell clearance in two independent models, WIN6 and TAM6. However, in the same syngeneic immunocompetent models, no *in vivo* benefit emerged when ALX301 was added to anti-GD2 or to a clinically aligned TEM/IRI plus anti-GD2 regimen.

No overt clinical or histopathological toxicity was observed under the conditions tested, although reductions in haemoglobin and haematocrit were detected at the CBC assessment. As CBCs were collected only at day 19 or humane endpoint, the kinetics of these haematological changes could not be determined, and we therefore cannot establish whether they were transient, progressive or recovering. Serial haematological monitoring would be required to more fully characterise the safety profile of ALX301 in combination with chemoimmunotherapy. Nevertheless, the absence of overt clinical signs of toxicity, including treatment-associated weight loss, together with the observation that mice reached humane endpoint because of tumour progression rather than treatment-related adverse effects, suggests that tolerability was unlikely to have been the principal limitation to efficacy under the conditions tested.

ALX301 was selected for *in vivo* evaluation because its Fc-silent design was anticipated to reduce the haematological toxicity associated with Fc-competent anti-CD47 antibodies while retaining CD47-blocking activity. The ALX301 dose and route of 30 mg/kg intraperitoneal injection used here were selected based on the provider’s guidance, and have previous activity in *in vivo* regimens across cancer models, including in a syngeneic neuroblastoma model [16,27]. However, pharmacokinetic measurements including circulating or intratumoural ALX301 concentrations and CD47 receptor occupancy were not assessed in the present study. We therefore cannot exclude inadequate tumour exposure or incomplete target engagement as contributors to the absence of detectable therapeutic benefit. The activity of the same ALX301 preparation in our *in vitro* assays confirms reagent functionality but does not establish *in vivo* exposure or tumour target engagement.

One potential explanation for the discrepancy between the *in vitro* and *in vivo* findings is that additional features of the TME may constrain macrophage-mediated tumour clearance. Untreated WIN6 and TAM6 tumours contained abundant myeloid populations, including MDSCs and M2-like macrophages, particularly in TAM6. It is therefore plausible that, in these models, interruption of the CD47 “Don’t Eat Me” signal alone may be insufficient to generate productive macrophage-mediated tumour clearance. However, because these immune profiles were obtained from untreated tumours collected outside the therapeutic experiments, the present data do not demonstrate that myeloid composition caused resistance to ALX301. Establishing whether treatment alters macrophage state, myeloid composition or antigen-presentation programmes, and whether these changes influence responsiveness to CD47 blockade, will require dedicated on-treatment and post-treatment profiling.

The 9464D-GD2 findings additionally demonstrate that resistance to macrophage-mediated phagocytosis can arise independently of the broader tumour microenvironment. Our *in vitro* analysis indicates that this resistance is not mediated by soluble factors and is not rescued by increasing the anti-GD2 dose, supporting the existence of tumour-cell-intrinsic determinants of susceptibility to macrophage-mediated clearance.

These findings contrast with the strong synergy reported by Theruvath and co-workers using the same anti-GD2 antibody (clone 14G2a) and ALX301 [16]. In that study, synergy was demonstrated in one syngeneic model derived from a different GEMM (*Th-MycN* in 129/SvJ mice) and in several human xenografts in immunodeficient hosts. Our results therefore indicate model-dependent heterogeneity: the regimen that was effective in those systems did not produce detectable benefit in WIN6 or TAM6 under the conditions tested. It remains possible that greater ALX301 exposure could provide benefit here; we did not escalate beyond the prolonged schedule in Fig. 7 because the dose and regimen used have documented activity and reflected provider guidance. In addition, ALX301 was the only CD47-targeting agent evaluated *in vivo* in the present study, and we did not assess whether alternative CD47-blocking agents might produce different therapeutic effects.

These observations are also relevant to the broader development of CD47-axis therapies, for which haematological on-target effects are well recognised while therapeutic activity has varied across disease settings and combination regimens [28,29]. Our findings support further investigation of the factors that determine responsiveness to CD47 blockade rather than identifying a specific resistance mechanism. One hypothesis arising from the baseline immune composition of WIN6 and TAM6 is that strategies that alter myeloid cell state could enhance responsiveness. There are several therapies already validated for preconditioning myeloid cells in the context of antitumour therapies, included are CSF1R blockade (to reduce suppressive macrophages) [30,31], CD40 agonists or STING/TLR agonists (to license APCs and skew macrophages) [32–34] or low-dose radiation (to boost “Eat Me” cues) [35]. Critically, future studies should incorporate longitudinal, on-treatment TME profiling alongside pharmacokinetic and target-engagement measurements to determine whether treatment modifies myeloid states and to establish whether these features are associated with sensitivity or resistance to CD47 blockade.

Strengths of this study include two immunocompetent, chemoresistant models with detailed immune contexture, clinically aligned dosing/schedules, matched *in vitro* and *in vivo* testing, and integrated safety readouts. Next steps should include on-treatment TME sampling and tests of myeloid-conditioning combinations with optimised scheduling; if tolerated, increased ALX301 exposure could also be explored. These efforts will clarify if, and under what conditions, CD47 blockade adds value in neuroblastoma.

Conclusion: In our neuroblastoma models, this ALX301 CD47 blockade agent, used in this regimen, does not augment anti-GD2 or chemoimmunotherapy *in vivo*, despite clear *in vitro* activity and acceptable safety. We propose that subsequent preclinical studies should test whether adding a myeloid-reprogramming agent to this regimen may create a pro-inflammatory macrophage niche to enable CD47 blockade to augment the efficacy of anti-GD2 chemoimmunotherapy.

## Supporting information

S1 Fig Flow cytometry gating strategy for detection of immune subpopulations. (PDF)

S2 Fig Dose titration of anti-GD2 in WIN6 and TAM6 tumour models. (PDF)

## Abbreviations

ACK: Ammonium-chloride-potassium
ADCC: Antibody-dependent cell cytotoxicity
ADCP: Antibody-dependent cellular phagocytosis
BMDM: Bone marrow derived macrophages
CBC: Complete blood count
CFSE: Carboxyfluorescein succinimidyl ester
CR: Complete response
GEMM: Genetically engineered mouse model
H&E: Haematoxylin and eosin
HED: Human-equivalent doses
IRI: Irinotecan
M-CSF: Macrophage colony-stimulating factor
n: Group size
NB: Neuroblastoma
PBS: Phosphate-buffered saline
RBCs: Red blood cells
SIRPα: Signal Regulatory Protein alpha
t-SNE: t-distributed stochastic neighbour embedding
TEM: Temozolomide
Th: Tyrosine hydroxylase
TME: Tumour microenvironment
