# Supplementary material for: CD47 blockade augments anti-GD2 driven phagocytosis in vitro but fails to improve in vivo efficacy in immunocompetent, chemoresistant neuroblastoma preclinical models
Source: PLoS One. 2026 Oct 27;21(10):e0353900. doi: 10.1371/journal.pone.0353900 (PMC13712761; doi:10.1371/journal.pone.0353900)
Supplement: Supplementary file 1 [file pone.0353900.s001.pdf]

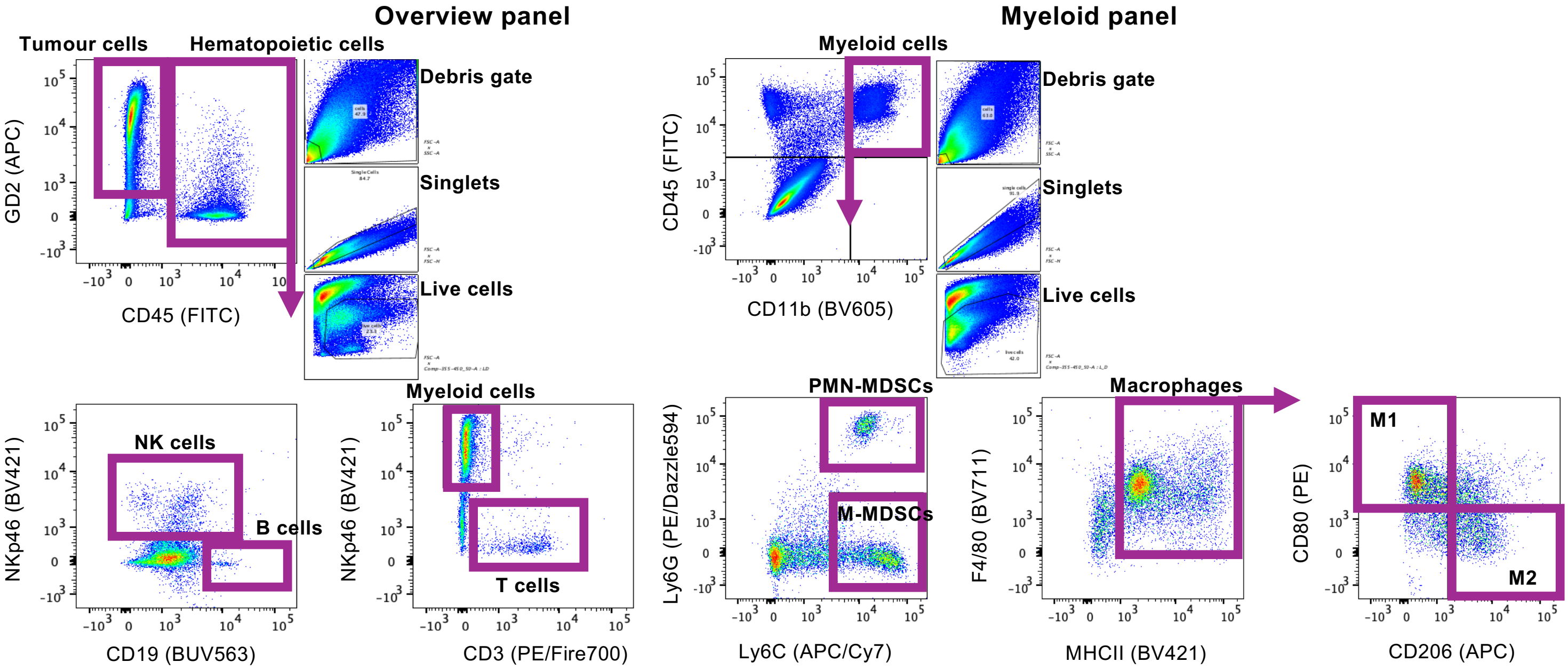

Supplementary figure 1. Flow cytometry gating strategy for detection of immune subpopulations.
a. *Representative gating strategy used to identify broad immune cell populations with an 'overview' panel and myeloid lineage cells with a 'myeloid' panel. Samples shown are on concatenated TAM6 tumours (n=5).*
