# Supplementary material for: CD47 blockade augments anti-GD2 driven phagocytosis in vitro but fails to improve in vivo efficacy in immunocompetent, chemoresistant neuroblastoma preclinical models
Source: PLoS One. 2026 Oct 27;21(10):e0353900. doi: 10.1371/journal.pone.0353900 (PMC13712761; doi:10.1371/journal.pone.0353900)
Supplement: Supplementary file 2 [file pone.0353900.s002.pdf]

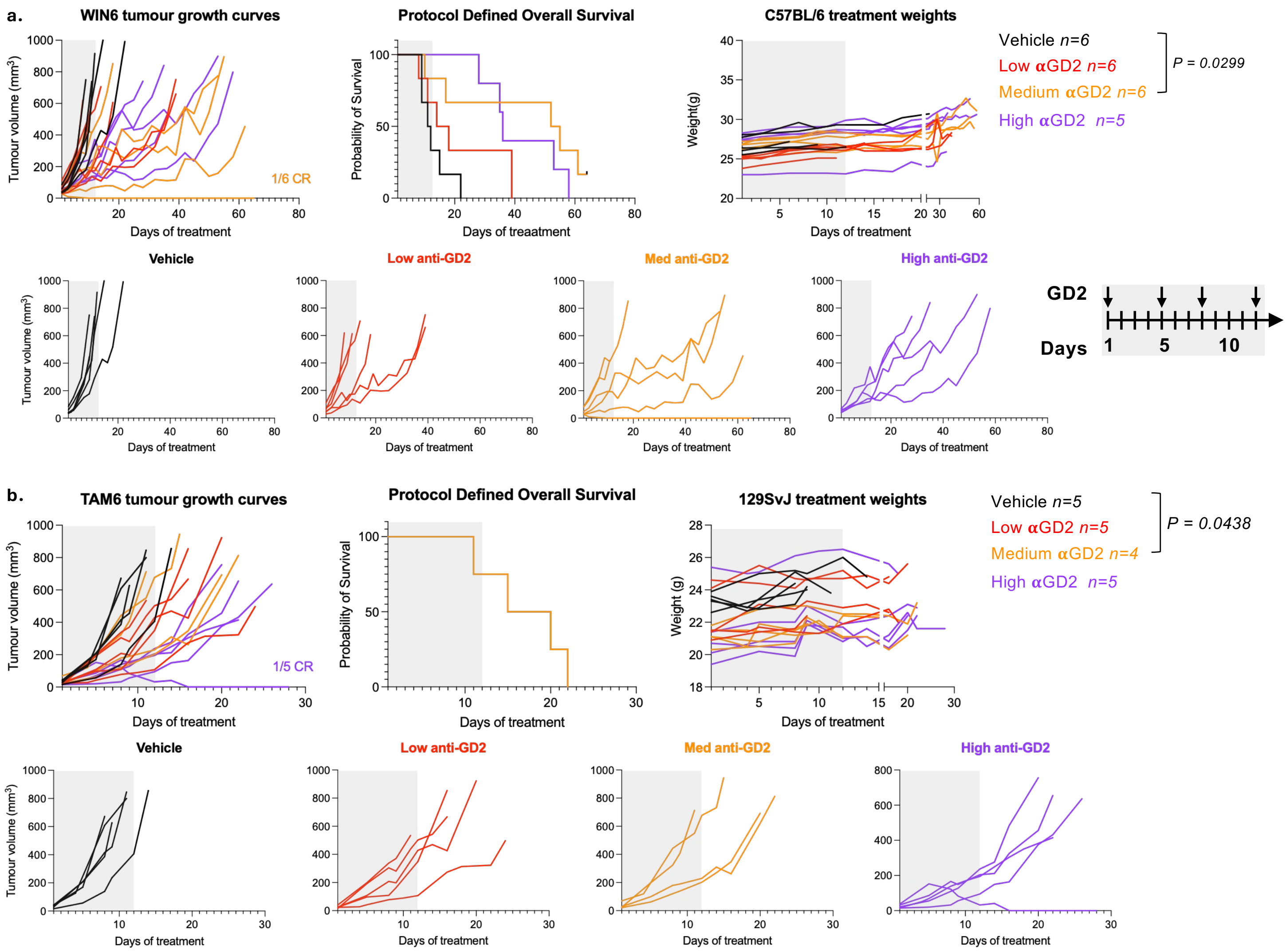

Supplementary figure 2. Dose titration of anti-GD2 in WIN6 and TAM6 tumour models.
a. *WIN6 tumour growth curves, survival, and treatment group weights following treatment with vehicle, low dose anti-GD2 (0.625 mg/kg), medium dose anti-GD2 (1.25 mg/kg) or high dose anti-GD2 (2.5 mg/kg). For the prespecified survival comparison of medium dose anti-GD2 versus vehicle, the hazard ratio was 0.1552 (95% CI 0.034-0.70); log-rank P = 0.0299).* ***b.*** *Equivalent experiment in TAM6 tumours, with treatment groups as indicated. For the prespecified survival comparison of medium dose anti-GD2 versus vehicle, the hazard ratio was 0.1286 (95% CI 0.02-0.78; log-rank P = 0.0438).*
