# Supplementary material for: CD47 blockade augments anti-GD2 driven phagocytosis in vitro but fails to improve in vivo efficacy in immunocompetent, chemoresistant neuroblastoma preclinical models
Source: PLoS One. 2026 Oct 27;21(10):e0353900. doi: 10.1371/journal.pone.0353900 (PMC13712761; doi:10.1371/journal.pone.0353900)
Supplement: Supplementary file 3 [file pone.0353900.s003.pdf]

ScreenIT Report

This is an automated report for 10.64898+2026.07.02.736004.pdf. This report was solicited by the PLOS One editorial team and provided by ScreenIT.

ScreenIT is an independent group of scientists developing automated tools that analyze academic papers. A set of automated tools screened your submitted manuscript and provided the report below. Each tool was created by your academic colleagues with the goal of helping authors.

The tools look for factors that are important for transparency, rigor and reproducibility, and we hope that the report might help you to improve reporting in your manuscript. Within the report you will find links to more information about the items that the tools check. These links include helpful papers, websites, or videos that explain why the item is important.

While our screening tools aim to improve and maintain quality standards they may, on occasion, miss nuances specific to your study type or flag something incorrectly. Each tool has limitations that are described on the ScreenIT website. The tools screen the main file for the paper; they are not able to screen supplements stored in separate files.

Please note that the Academic Editor had access to these comments while making a decision on your manuscript. The Academic Editor may ask that issues flagged in this report be addressed.

If you would like to provide feedback on the ScreenIT tool, please email the team at ScreenIt@bih-charite.de. If you have questions or concerns about the review process, please contact the PLOS One office at plosone@plos.org.

How to read the report:

- no action required
- review for potential issues
- a critical issue was found by a tool
- may not be applicable to your paper

- more information about this item can be obtained using this link

Rigor

?	Ethics	not detected.
✓	Inclusion and Exclusion Criteria	"In total, 187 mice were engrafted and 115 enrolled and analysed; no treated animals were excluded from analysis."
?	Flow charts and attrition	We did not find a study flow chart of excluded observations. We strongly recommend using flow charts because they provide an overview of the study design and more information about attrition. If you included a study flow chart in your supplemental files, we apologize for missing this. Our tool is not able to screen separate supplemental files.<br><br>Sentence about attrition: "not required."
✓	Sex as a biological variable	"TAM6 was derived from a tumour in a female mouse and WIN6 from a male mouse; therefore all subsequent allografts were sex-matched within their respective strains to avoid confounding immune responses to sex-linked (e.g., H-Y) antigens."
✓	Subject Demographics	Age: "Mice of 8-12 weeks old were tumour engrafted."
✓	Randomization	"Animals were ranked by baseline volume, binned from smallest to largest, and randomly assigned to groups via a list generator to balance means/SDs across arms."
✓	Blinding	"Assessments for euthanasia and calliper measurements were performed by investigators blinded to treatment allocation."
✓	Power Analysis	"No formal a priori power calculation was performed because effect sizes in these models were unknown."
✓	Replication	"Group sizes (n = 58/arm) were chosen from prior variability and 3Rs principles to detect large, practicechanging effects; key null findings were replicated in independent cohorts and in both models."

Generated by Barzooka (flow charts), SciScore (everything else).
SciScore only screens the text of the methods section; we apologize if the tool has missed items reported elsewhere in the paper or supplements.

Resources

Persistent unique identifiers for research resources, RRIDs, can be used to track these key resources though the literature. They are requested or required in many journals, but even if your journal does not require them, we strongly encourage the use of these for the readers of papers who wish to easily track down the key materials like antibodies, software tools, or mice.

Antibodies	"Temozolomide (TEM), irinotecan (IRI), and anti-GD2 monoclonal antibody (clone 14G2a, Bio X Cell) were used at clinically relevant levels."	TEM suggested: None. Please add an RRID to help others identify this resource.<br>anti-GD2 suggested: None. Please add an RRID to help others identify this resource.
Antibodies	"Agents were added as: anti-GD2 antibody alone (0-100nM), CD47 blocker alone (anti-CD47 or ALX301, 0-100nM), or CD47 blocker (0-100nM) plus suboptimal anti-GD2 (0.5nM)."	anti-CD47 suggested: None. Please add an RRID to help others identify this resource.<br>ALX301 suggested: None. Please add an RRID to help others identify this resource.
Experimental Models: Organisms/ Strains	"Spontaneous tumours from the ThMycN+/-/Th-AlkF1147L C57Bl/6 mice were allografted subcutaneously and treated intraperitoneally with a single cycle of VAC chemotherapy calculated at clinically relevant doses: 0.0015mg/kg vincristine, 0.68mg/ kg doxorubicin and	C57Bl/6 suggested: None. Please add an RRID to help others identify this resource.

	32mg/kg cyclophosphamide."	
Experimental Models: Organisms/ Strains	"19)); 5x10 cells/ mouse for WIN6 yielded consistent growth in C57BL/6J mice."	C57BL/6J suggested: RRID:IMSR_JAX:000664. Please make sure this RRID suggestion is accurate before including in your manuscript.
Software and Algorithms	"Analysis was performed in FlowJo."	FlowJo suggested: FlowJo, RRID:SCR_008520. Please make sure this RRID suggestion is accurate before including in your manuscript.
Software and Algorithms	"Data was processed in Microsoft Excel and graphed in GraphPad Prism."	Microsoft Excel suggested: Microsoft Excel, RRID:SCR_016137. Please make sure this RRID suggestion is accurate before including in your manuscript.<br>GraphPad Prism suggested: GraphPad Prism (RRID:SCR_002798. Please make sure this RRID suggestion is accurate before including in your manuscript.

Generated by SciScore.
SciScore only screens the text of the methods section; we apologize if the tool has missed items reported elsewhere in the paper or supplements.

Transparency

?	Open code	If you wrote code to analyze your data, please consider sharing the code in a public repository. This makes it easier for others to reproduce your analyses, and may also aid others seeking to analyze similar datasets.
?	Open data	If permitted, sharing data on a public repository with a persistent identifier (a DOI or accession number) can improve reproducibility and make it easier for other scientists to expand on your work. Papers with open data are cited more often than papers without open data. Some institutions have an expert who can provide advice on data sharing.

?	Self-acknowledged limitations	An explicit section about the limitations of this study was not found. We encourage authors to include a paragraph in the discussion that addresses study limitations. Every study has limitations. Describing these limitations helps readers to understand and contextualize the research.
?	Conflict of interest statement	It is important to supply a conflict of interest statement, even if there are none to declare, to transparently present the research.
✓	Funding statement	Thank you for reporting your funding source(s), or stating that there was no specific funding for this work.
n/a	Registration statement	A registered protocol makes the planned research project more transparent and can protect against bias. Please consider registering future confirmatory studies.

Generated by ODDPub, limitation-recognizer, rtransparent, trial-identifier.

Figures

?	Bar graphs of continuous data	We found bar graphs of continuous data. We recommend replacing these with more informative graphics (e.g. dotplots, box plots, violin plots), as many different datasets can lead to the same bar graph. The actual data may suggest different conclusions from the summary statistics alone. Please see the following resources for more information on the problems with bar graphs (Weissgerber et al 2015) and what to use instead (Weissgerber et al 2019).
?	Bar graphs with dot plots	Thank you for showing individual data points on your bar graphs. We recommend removing the bars and showing only a dot plot. The resource linked in the information symbol illustrates several reasons why dot plots are better than bar graphs with dot plots.
✓	No rainbow color maps	Thank you for not using rainbow colormaps. Rainbow color maps distort readers' perception of the data by introducing visual artifacts and are not accessible to colorblind readers.

Generated by JetFighter, Barzooka.
