# Supplementary material for: CD47 blockade augments anti-GD2 driven phagocytosis in vitro but fails to improve in vivo efficacy in immunocompetent, chemoresistant neuroblastoma preclinical models
Source: PLoS One. 2026 Oct 27;21(10):e0353900. doi: 10.1371/journal.pone.0353900 (PMC13712761; doi:10.1371/journal.pone.0353900)
Supplement: Supplementary file 4 [file pone.0353900.s005.docx]

Response to Reviewers

We sincerely thank the reviewer for the detailed and constructive assessment of our manuscript. We have carefully considered each point raised and have made substantial revisions throughout the manuscript, with individual responses provided below.

We particularly appreciate the reviewer’s recognition that the establishment and immune characterisation of the TAM6 and WIN6 models represents a contribution beyond the specific therapeutic findings reported here. We also agree that dissemination of well-controlled negative preclinical findings is important, particularly for therapeutic strategies that are being actively considered for clinical translation, as is the case for CD47-targeted immunotherapy.

In response to the reviewer’s central concerns, we have strengthened the quantitative reporting of the in vivo studies by adding hazard ratios, 95% confidence intervals and corresponding statistical comparisons for the key efficacy analyses. We have also substantially revised the Discussion to more clearly distinguish conclusions directly supported by the present data from hypotheses arising from those findings. In particular, we now explicitly acknowledge the absence of pharmacokinetic and target-engagement measurements, the lack of on-treatment or post-treatment tumour microenvironment profiling and the limitations of the toxicity assessment.

More broadly, mechanistic interpretations have been revised throughout to use appropriately conditional language, and proposed explanations for the lack of in vivo efficacy are now presented as hypotheses requiring further investigation rather than as established mechanisms.

We believe these revisions have substantially strengthened the manuscript and aligned the conclusions more closely with the evidence presented. We hope that the revised version now addresses the reviewer’s concerns in full.

Comments
#1. The manuscript concludes that ALX301 does not improve efficacy in vivo, but no effect size, hazard ratio, or confidence interval is reported for any of the key in vivo comparisons in Figures 5, 6, or 7. Reporting only that a comparison is not significant is not sufficient to support a negative claim of this kind. Without a quantified effect size and its confidence interval, it is not possible for the reader to distinguish a true absence of effect from an underpowered study that simply failed to detect a real but modest effect. This is especially important given that no a priori power calculation was performed, as the authors themselves acknowledge.

We thank the reviewer for this important suggestion, which has strengthened the quantitative support for the central negative finding of the manuscript. We have now added effect estimates for the key in vivo survival comparisons in Figures 5-7. Hazard ratios with 95% confidence intervals, together with the corresponding log-rank P values, are reported in the revised figure legends.

We have also added the relevant P values directly to the main survival figures in Figures 5-7, specifically for the treatment-group comparisons being evaluated in the manuscript. The same approach has been applied to Supplementary Figure 2, where the anti-GD2 dose-finding experiment is shown.

In Supplementary Figure 2, the medium-dose anti-GD2 group demonstrated a significant survival benefit compared with vehicle, supporting selection of this dose for the subsequent combination studies with ALX301.

For the reviewer’s convenience, these additions have been highlighted within the revised figure legends in the marked-up manuscript on pages 29-30 for Figures 5-7 and page 31 for Supplementary Figure 2.

#2. There is no pharmacokinetic or pharmacodynamic data supporting that ALX301 achieved adequate exposure or target engagement in vivo. No blood or tumor concentration data, half life, or receptor occupancy data for CD47 are provided. The statement that the same reagent lot was used in vitro and in vivo does not address this concern, since lot consistency does not establish that the drug reached the tumor at a concentration sufficient to block CD47 in that tissue. Without this evidence, the negative in vivo result cannot be distinguished from a dosing or exposure failure rather than a true biological lack of efficacy.

We thank the reviewer for this important point and agree that greater emphasis is required in the manuscript to point the reader to the lack of definitively proven failure of therapeutic effect of ALX301 in vivo.

We have therefore revised the fourth paragraph of the Discussion to explicitly acknowledge this limitation. Moreover, we have also clarified later in the same paragraph that activity of the same ALX301 preparation in our in vitro assays demonstrates reagent functionality only and does not establish adequate in vivo exposure or tumour target engagement. We include the following text (pages 20-21 highlighted):

*“Pharmacokinetic measurements including circulating or intratumoural ALX301 concentrations and CD47 receptor occupancy were not assessed in the present study. We therefore cannot exclude inadequate tumour exposure or incomplete target engagement as contributors to the absence of detectable therapeutic benefit. The activity of the same ALX301 preparation in our in vitro assays confirms reagent functionality but does not establish in vivo exposure or tumour target engagement.”*

#3. The Discussion attributes the lack of in vivo benefit to a myeloid rich, immunosuppressive tumor microenvironment, but this characterization is based exclusively on untreated tumors collected outside the treatment window, as stated in the Methods. No on treatment or post treatment tumor microenvironment data are presented. The manuscript should either tone down this mechanistic claim considerably, using more conditional language throughout, or acknowledge more clearly that this is a hypothesis for future testing rather than a finding supported by the present data.

We thank the reviewer for this important point and agree that the mechanistic interpretation should be more clearly qualified and presented as a hypothesis for future investigation rather than as a conclusion established by the present data.

Accordingly, we have substantially revised the Discussion, as well as related wording throughout the manuscript, to avoid presenting the baseline myeloid-rich tumour microenvironment as a definitive mechanism of resistance to ALX301.

In the fifth paragraph of the Discussion (page 21 line 424), we now describe the tumour microenvironment as a potential explanation for the lack of in vivo efficacy and explicitly acknowledge that the immune profiling was performed on untreated tumours collected outside the therapeutic experiments. We therefore state that the present data do not prove that myeloid composition caused resistance to ALX301.

Later in the same paragraph, we also explicitly acknowledge that no on-treatment or post-treatment tumour microenvironment profiling was performed. The revised Discussion now emphasises that dedicated longitudinal profiling will be required to determine whether treatment alters macrophage state, myeloid composition or antigen-presentation programmes, and whether these features are associated with responsiveness to CD47 blockade.

For the reviewer’s convenience, these revisions have been highlighted in the marked-up manuscript.

#4. Toxicity was assessed using a single complete blood count timepoint at day 19 or at humane endpoint. A single timepoint cannot establish whether the observed reduction in hemoglobin and hematocrit is transient or progressive. A time course including earlier timepoints, for example days 3, 7, and 14, would be needed to properly characterize the safety profile of ALX301 in combination with chemoimmunotherapy, particularly since red cell effects are described as an expected, on target consequence of CD47 blockade that could plausibly recover or worsen with continued dosing.

We thank the reviewer for this important comment and agree that a single blood count timepoint cannot determine whether the observed reductions in haemoglobin and haematocrit were transient, progressive or recovering over time.

We have therefore revised the third paragraph of the discussion (page 20 line 400) to state this explicitly as a limitation. We now clarify that blood was collected only at day 19 or at humane endpoint, that the kinetics of these haematological changes cannot be determined from the present study, and that serial haematological monitoring would be required to more fully characterise the safety profile of ALX301 in combination with chemoimmunotherapy.

We have also moderated the interpretation of the safety data accordingly (page 18 line 366). While no overt clinical or histopathological toxicity was observed under the conditions tested, we no longer present the available blood count data as sufficient to establish the temporal profile of treatment-related haematological effects.

For the reviewer’s convenience, these revisions have been highlighted in the marked-up manuscript.

#5. Several in vivo cohorts are very small, for example n equals 3 for the TAM6 vehicle and combination groups in Figure 5b. Combined with the absence of a power calculation, this raises the concern that the study may simply be underpowered to detect a real effect in this particular model, rather than providing strong evidence that no effect exists. The authors should at minimum discuss the limitations this places on interpretation of the TAM6 findings specifically.

Thank you for highlighting the small group sizes and the resulting limitations in statistical power. In response to this comment, we performed a thorough re-review of the raw in vivo data and identified that several treated animals had previously been excluded from the plotted analyses. On reassessment, we determined that the exclusion criterion applied to these animals was not appropriate. We have therefore revised the analyses to include all treated animals enrolled in these experiments.

This has increased the group sizes in the affected cohorts, and the corresponding figures statistical analyses and underlying source data have been updated accordingly (Figure 5b, legend page 29-30). Importantly, inclusion of these animals did not alter the overall conclusions of the experiments, but provides a more complete representation of the treated cohorts.

The Methods have also been corrected to state explicitly that all treated animals enrolled in the studies are included in the final analysis (page 10 line 316).

#6. Blinding is explicitly described only for humane endpoint assessment and calliper based tumor measurement. It is not stated whether drug administration, complete blood count analysis, or histopathological scoring of heart, lung, kidney, liver, spleen, and tumor sections were performed with the investigator blinded to treatment allocation. Given that the histopathology conclusion of no tissue injury and the hematology comparisons both involve an element of interpretation, blinding status for these assessments should be clarified.

We thank the reviewer for requesting clarification regarding blinding.

Drug administration was not performed blinded to treatment allocation. A practical limitation was that temozolomide and irinotecan were diluted in PBS immediately prior to administration to minimise the risk of precipitation or loss of solubility, meaning that treatment syringes could not be prepared substantially in advance and independently relabelled for blinded dosing. We have clarified this in the Methods under In vivo studies and treatment combinations (highlighted on page 10 line 190-192).

Importantly, tumour measurements and humane endpoint assessments were performed blinded to treatment allocation, thereby reducing the potential for treatment knowledge to influence the primary efficacy assessments (Methods page 12 line 401).

Complete blood count analysis was performed externally by IDEXX UK. Histological assessment was not performed using a formal blinded scoring system. We have clarified both points in the Methods (page 11 line 373).

We have also made clear in the Methods section entitled Blood collection and histology (page 11 line 360) that the histological assessment was qualitative, that no formal histopathological scoring was performed, and that sections were not independently reviewed by a veterinary pathologist. Accordingly, we have moderated the wording in the discussion so that the absence of overt histological abnormalities is presented as supportive evidence of tolerability rather than as definitive evidence of an absence of tissue toxicity (page 20 from line 756).

#7. The selection of ALX301 as the sole CD47 blocking agent tested in vivo is justified in the Discussion primarily on the basis of an anticipated favorable toxicity profile relative to Fc competent anti CD47 antibodies. Since this is the only in vivo agent tested and no in vivo dose escalation beyond the schedule used was performed, the rationale for fixing both the agent and the dose at a single level determined by provider guidance should be stated more explicitly, and the implications of not testing higher exposure should be discussed as a clear limitation rather than mentioned only briefly.

We have now explicitly noted in the results that the complete response rate was numerically lower in the chemoimmunotherapy plus ALX301 group than in the chemoimmunotherapy backbone group (4/7 versus 5/7, respectively), although this was not accompanied by a significant survival difference. This addition has been highlighted in the Results section entitled “Intensified CD47 blockade with chemoimmunotherapy showed favourable safety profile but no additional benefit.” (highlighted on page 18 lines 362-265).

We have also taken this comment into account more broadly when revising the manuscript and have ensured that potentially relevant differences suggesting reduced efficacy with ALX301 are reported and appropriately contextualised. For example, in the WIN6 anti-GD2 monotherapy experiment, the anti-GD2-only group showed a significant survival advantage over the anti-GD2 plus ALX301 group, including one complete response. We now report this finding explicitly, while also noting that it was not reproduced in TAM6 or in subsequent experiments and therefore does not provide consistent evidence that ALX301 antagonises anti-GD2 activity. This addition has been highlighted in the Results section entitled “CD47 blockade does not improve anti-GD2 antibody or chemoimmunotherapy *in vivo*.” (page 17 line 333).

#9. Several passages in the Discussion use assertive, declarative language, for example stating that the findings are suggesting that the myeloid niche limits benefit, when the underlying evidence is associative rather than mechanistic. The authors should revise this language throughout to more conditional wording such as may or could, so that the strength of the claims matches the strength of the supporting data.

As also addressed in response to Comment #3, we have revised the Discussion extensively throughout to ensure that the strength of the language more appropriately reflects the associative nature of the supporting data.

In particular, proposed explanations relating to the tumour microenvironment are now presented using conditional language and framed as hypotheses for future investigation rather than as mechanisms directly demonstrated by the present study. We have also revised related wording elsewhere in the manuscript to ensure that conclusions remain aligned with the strength of the available evidence.

#10. The Data Availability Statement indicates only that data will be made available upon acceptance, without specifying a repository or identifier. A more specific statement identifying where the minimal dataset will be deposited would strengthen compliance with data sharing expectations.

A full data package has been prepared for upload and will be deposited in Open Science Framework upon manuscript acceptance.

#11. There are several recurring typographical errors that should be corrected throughout, including Institute of Chid Health, which should read Institute of Child Health, and Research into Chidhood Cancer, which should read Research into Childhood Cancer, along with buffer and from, which appear as bufffer and ffrom in the Methods.

Thank you for such careful reading of the manuscript. We have carefully proofread it and corrected the specific errors identified, including “Institute of Child Health”, “Research into Childhood Cancer”, “buffer” and “from”, as well as other typographical inconsistencies identified throughout the text.

#12. The schematic in Figure 1a states that the N297A mutation renders the Fc region of ALX301 inactive. This is not entirely accurate, since N297A abrogates Fc gamma receptor engagement specifically while the CD47 binding function of the SIRP alpha domain is preserved. The figure legend or label should be revised for precision.

We have revised the Figure 1a annotation adjacent to the Fc portion of ALX301 to describe the effect of the N297A mutation more precisely. ALX301 is now described as comprising an engineered SIRPα D1 domain fused to a mouse IgG1 Fc containing the N297A mutation, which abrogates Fcγ receptor engagement while preserving CD47 binding. This replaces the previous, less precise description of the Fc region as “inactive”.

#13. Reference 20 lists author initials only, for example T B, W L, N B, rather than full author names, and should be corrected to a standard citation format.

Thanks for pointing this out. The reference has been corrected to include the appropriate author names and initials rather than initials alone. We have also reviewed the complete reference list and corrected additional inconsistencies.
