# Supplementary material for: CD47 blockade augments anti-GD2 driven phagocytosis in vitro but fails to improve in vivo efficacy in immunocompetent, chemoresistant neuroblastoma preclinical models
Source: PLoS One. 2026 Oct 27;21(10):e0353900. doi: 10.1371/journal.pone.0353900 (PMC13712761; doi:10.1371/journal.pone.0353900)
Supplement: Peer review history

# Peer review: Decision Letter 0

**DOI:** 10.1371/journal.pone.0353900.r001

**Authors:** Lu Zhang

23 Jul 2026

Dear Dr. Himsworth,

Thank you for submitting your manuscript to PLOS ONE. After careful consideration, we feel that it has merit but does not fully meet PLOS ONE’s publication criteria as it currently stands. Therefore, we invite you to submit a revised version of the manuscript that addresses the points raised during the review process.

Please submit your revised manuscript by Sep 21 2026 11:59PM. If you will need more time than this to complete your revisions, please reply to this message or contact the journal office at plosone@plos.org. When you're ready to submit your revision, log on to https://www.editorialmanager.com/pone/ and select the 'Submissions Needing Revision' folder to locate your manuscript file.

- A letter that responds to each point raised by the academic editor and reviewer(s). You should upload this letter as a separate file labeled 'Response to Reviewers'.
- A marked-up copy of your manuscript that highlights changes made to the original version. You should upload this as a separate file labeled 'Revised Manuscript with Track Changes'.
- An unmarked version of your revised paper without tracked changes. You should upload this as a separate file labeled 'Manuscript'.

If you would like to make changes to your financial disclosure, please include your updated statement in your cover letter. Guidelines for resubmitting your figure files are available below the reviewer comments at the end of this letter.

If applicable, we recommend that you deposit your laboratory protocols in protocols.io to enhance the reproducibility of your results. Protocols.io assigns your protocol its own identifier (DOI) so that it can be cited independently in the future. For instructions see: https://journals.plos.org/plosone/s/submission-guidelines#loc-laboratory-protocols. Additionally, PLOS ONE offers an option for publishing peer-reviewed Lab Protocol articles, which describe protocols hosted on protocols.io. Read more information on sharing protocols at https://plos.org/protocols?utm_medium=editorial-email&utm_source=authorletters&utm_campaign=protocols.

As the corresponding author, your ORCID iD is verified in the submission system and will appear in the published article. PLOS supports the use of ORCID, and we encourage all coauthors to register for an ORCID iD and use it as well. Please encourage your coauthors to verify their ORCID iD within the submission system before final acceptance, as unverified ORCID iDs will not appear in the published article. *Only*  the individual author can complete the verification step; PLOS staff *cannot*  verify ORCID iDs on behalf of authors.

We look forward to receiving your revised manuscript.

Kind regards,

Lu Zhang

Academic Editor

PLOS One

Journal Requirements:

When submitting your revision, we need you to address these additional requirements.

1. Please ensure that your manuscript meets PLOS ONE's style requirements, including those for file naming. The PLOS ONE style templates can be found at

https://journals.plos.org/plosone/s/file?id=wjVg/PLOSOne_formatting_sample_main_body.pdf and

https://journals.plos.org/plosone/s/file?id=ba62/PLOSOne_formatting_sample_title_authors_affiliations.pdf

2. Thank you for stating the following in the Acknowledgments Section of your manuscript:

“We would like to acknowledge grant support from Solving Kids Cancer, National institute of Health Research, Little Princess Trust, Cancer Research UK Grand Challenge, SU2C, Great Ormond Street Hosp Biomedical Research Centre and Research into Chidhood Cancer (RICC).”

We note that you have provided funding information that is not currently declared in your Funding Statement. However, funding information should not appear in the Acknowledgments section or other areas of your manuscript. We will only publish funding information present in the Funding Statement section of the online submission form.

Please remove any funding-related text from the manuscript and let us know how you would like to update your Funding Statement. Currently, your Funding Statement reads as follows:

“Solving Kids Cancer: Optimizing combination immunotherapy for primary chemorefractory neuroblastoma. PI John Anderson, 572532: £240,000 Research In CHildhood Cancer (RICC); core support; PI John Anderson: 502915: £65,000 NIHR Great Ormond Street Biomedical Research Centre PI John ANderson”

Please include your amended statements within your cover letter; we will change the online submission form on your behalf.

3. We note that the grant information you provided in the ‘Funding Information’ and ‘Financial Disclosure’ sections do not match.

When you resubmit, please ensure that you provide the correct grant numbers for the awards you received for your study in the ‘Funding Information’ section.

4. Thank you for stating the following financial disclosure:

“Solving Kids Cancer: Optimizing combination immunotherapy for primary chemorefractory neuroblastoma. PI John Anderson, 572532: £240,000 Research In CHildhood Cancer (RICC); core support; PI John Anderson: 502915: £65,000 NIHR Great Ormond Street Biomedical Research Centre PI John ANderson”

Please state what role the funders took in the study.  If the funders had no role, please state: "The funders had no role in study design, data collection and analysis, decision to publish, or preparation of the manuscript."

If this statement is not correct you must amend it as needed.

Please include this amended Role of Funder statement in your cover letter; we will change the online submission form on your behalf.

5. When completing the data availability statement of the submission form, you indicated that you will make your data available on acceptance. We strongly recommend all authors decide on a data sharing plan before acceptance, as the process can be lengthy and hold up publication timelines. Please note that, though access restrictions are acceptable now, your entire data will need to be made freely accessible if your manuscript is accepted for publication. This policy applies to all data except where public deposition would breach compliance with the protocol approved by your research ethics board. If you are unable to adhere to our open data policy, please kindly revise your statement to explain your reasoning and we will seek the editor's input on an exemption. Please be assured that, once you have provided your new statement, the assessment of your exemption will not hold up the peer review process.

6. Please include captions for your Supporting Information files at the end of your manuscript, and update any in-text citations to match accordingly. Please see our Supporting Information guidelines for more information: http://journals.plos.org/plosone/s/supporting-information.

7. If the reviewer comments include a recommendation to cite specific previously published works, please review and evaluate these publications to determine whether they are relevant and should be cited. There is no requirement to cite these works unless the editor has indicated otherwise.

[Note: HTML markup is below. Please do not edit.]

Reviewers' comments:

Reviewer's Responses to Questions

**Comments to the Author**

1. Is the manuscript technically sound, and do the data support the conclusions?

Reviewer #1: Yes

Reviewer #2: No

**********

2. Has the statistical analysis been performed appropriately and rigorously?

Reviewer #1: Yes

Reviewer #2: No

**********

3. Have the authors made all data underlying the findings in their manuscript fully available?

The PLOS Data policy

Reviewer #1: Yes

Reviewer #2: No

**********

4. Is the manuscript presented in an intelligible fashion and written in standard English?

Reviewer #1: Yes

Reviewer #2: Yes

**********

Reviewer #1: This is an automated report for PONE-D-26-32803. This report was solicited by the PLOS One editorial team and provided by ScreenIT. ScreenIT is an independent group of scientists developing automated tools that analyze academic papers. A set of automated tools screened your submitted manuscript and provided the report below. Each tool was created by your academic colleagues with the goal of helping authors. The tools look for factors that are important for transparency, rigor and reproducibility, and we hope that the report might help you to improve reporting in your manuscript. Within the report you will find links to more information about the items that the tools check. These links include helpful papers, websites, or videos that explain why the item is important. While our screening tools aim to improve and maintain quality standards they may, on occasion, miss nuances specific to your study type or flag something incorrectly. Each tool has limitations that are described on the ScreenIT website. The tools screen the main file for the paper; they are not able to screen supplements stored in separate files. Please note that the Academic Editor had access to these comments while making a decision on your manuscript. The Academic Editor may ask that issues flagged in this report be addressed. If you would like to provide feedback on the ScreenIT tool, please email the team at ScreenIt@bih-charite.de. If you have questions or concerns about the review process, please contact the PLOS One office at plosone@plos.org.

Reviewer #2: Thank you for the opportunity to review this manuscript.

This manuscript reports that a Fc silent CD47 blocker, ALX301, augments anti GD2 driven phagocytosis in vitro in two newly characterized immunocompetent, chemoresistant neuroblastoma models, TAM6 and WIN6, but fails to translate into improved tumor control or survival in vivo when combined with anti GD2 monotherapy or with a temozolomide/irinotecan chemoimmunotherapy backbone. Toxicity was limited to mild, expected red cell changes. The establishment and immune characterization of the TAM6 and WIN6 models is a genuine contribution and the willingness to report a negative combination result is commendable. However, the manuscript in its current form does not provide sufficient quantitative and mechanistic support for its central negative claim, and several aspects of the experimental design and statistical reporting need to be substantially strengthened before the conclusions can be considered fully supported by the data. In particular, the paper reports absence of efficacy without reporting effect sizes or confidence intervals for the key comparisons, without any pharmacokinetic or target engagement data for ALX301 in vivo, and without post treatment tumor microenvironment data to support the mechanistic explanation offered in the Discussion. Toxicity assessment is also based on a single timepoint rather than a longitudinal profile, and blinding is only explicitly stated for endpoint assessment and calliper measurement, not for drug administration, hematology, or histopathology scoring. Taken together, these issues mean that the manuscript's negative conclusion, while plausible, is not yet fully substantiated by the data presented, and the Discussion in several places extends beyond what the data can support.

Comments

#1. The manuscript concludes that ALX301 does not improve efficacy in vivo, but no effect size, hazard ratio, or confidence interval is reported for any of the key in vivo comparisons in Figures 5, 6, or 7. Reporting only that a comparison is not significant is not sufficient to support a negative claim of this kind. Without a quantified effect size and its confidence interval, it is not possible for the reader to distinguish a true absence of effect from an underpowered study that simply failed to detect a real but modest effect. This is especially important given that no a priori power calculation was performed, as the authors themselves acknowledge.

#2. There is no pharmacokinetic or pharmacodynamic data supporting that ALX301 achieved adequate exposure or target engagement in vivo. No blood or tumor concentration data, half life, or receptor occupancy data for CD47 are provided. The statement that the same reagent lot was used in vitro and in vivo does not address this concern, since lot consistency does not establish that the drug reached the tumor at a concentration sufficient to block CD47 in that tissue. Without this evidence, the negative in vivo result cannot be distinguished from a dosing or exposure failure rather than a true biological lack of efficacy.

#3. The Discussion attributes the lack of in vivo benefit to a myeloid rich, immunosuppressive tumor microenvironment, but this characterization is based exclusively on untreated tumors collected outside the treatment window, as stated in the Methods. No on treatment or post treatment tumor microenvironment data are presented. The manuscript should either tone down this mechanistic claim considerably, using more conditional language throughout, or acknowledge more clearly that this is a hypothesis for future testing rather than a finding supported by the present data.

#4. Toxicity was assessed using a single complete blood count timepoint at day 19 or at humane endpoint. A single timepoint cannot establish whether the observed reduction in hemoglobin and hematocrit is transient or progressive. A time course including earlier timepoints, for example days 3, 7, and 14, would be needed to properly characterize the safety profile of ALX301 in combination with chemoimmunotherapy, particularly since red cell effects are described as an expected, on target consequence of CD47 blockade that could plausibly recover or worsen with continued dosing.

#5. Several in vivo cohorts are very small, for example n equals 3 for the TAM6 vehicle and combination groups in Figure 5b. Combined with the absence of a power calculation, this raises the concern that the study may simply be underpowered to detect a real effect in this particular model, rather than providing strong evidence that no effect exists. The authors should at minimum discuss the limitations this places on interpretation of the TAM6 findings specifically.

#6. Blinding is explicitly described only for humane endpoint assessment and calliper based tumor measurement. It is not stated whether drug administration, complete blood count analysis, or histopathological scoring of heart, lung, kidney, liver, spleen, and tumor sections were performed with the investigator blinded to treatment allocation. Given that the histopathology conclusion of no tissue injury and the hematology comparisons both involve an element of interpretation, blinding status for these assessments should be clarified.

#7. The selection of ALX301 as the sole CD47 blocking agent tested in vivo is justified in the Discussion primarily on the basis of an anticipated favorable toxicity profile relative to Fc competent anti CD47 antibodies. Since this is the only in vivo agent tested and no in vivo dose escalation beyond the schedule used was performed, the rationale for fixing both the agent and the dose at a single level determined by provider guidance should be stated more explicitly, and the implications of not testing higher exposure should be discussed as a clear limitation rather than mentioned only briefly.

#8. In Figure 7a, the chemoimmunotherapy plus ALX301 group shows a numerically lower complete response rate than the chemoimmunotherapy alone group, 4 of 7 versus 5 of 7. This trend, while likely not statistically significant given the sample size, is not mentioned anywhere in the Results or Discussion. Given that this is the only figure in the manuscript combining chemotherapy, anti GD2, and ALX301 with survival data, the authors should explicitly comment on this observation rather than omitting it.

#9. Several passages in the Discussion use assertive, declarative language, for example stating that the findings are suggesting that the myeloid niche limits benefit, when the underlying evidence is associative rather than mechanistic. The authors should revise this language throughout to more conditional wording such as may or could, so that the strength of the claims matches the strength of the supporting data.

#10. The Data Availability Statement indicates only that data will be made available upon acceptance, without specifying a repository or identifier. A more specific statement identifying where the minimal dataset will be deposited would strengthen compliance with data sharing expectations.

#11. There are several recurring typographical errors that should be corrected throughout, including Institute of Chid Health, which should read Institute of Child Health, and Research into Chidhood Cancer, which should read Research into Childhood Cancer, along with buffer and from, which appear as bufffer and ffrom in the Methods.

#12. The schematic in Figure 1a states that the N297A mutation renders the Fc region of ALX301 inactive. This is not entirely accurate, since N297A abrogates Fc gamma receptor engagement specifically while the CD47 binding function of the SIRP alpha domain is preserved. The figure legend or label should be revised for precision.

#13. Reference 20 lists author initials only, for example T B, W L, N B, rather than full author names, and should be corrected to a standard citation format.

**********

what does this mean?). If published, this will include your full peer review and any attached files.

If you choose “no”, your identity will remain anonymous but your review may still be made public.

**Do you want your identity to be public for this peer review?**  For information about this choice, including consent withdrawal, please see our Privacy Policy

Reviewer #1: No

Reviewer #2: No

**********

[NOTE: If reviewer comments were submitted as an attachment file, they will be attached to this email and accessible via the submission site. Please log into your account, locate the manuscript record, and check for the action link "View Attachments". If this link does not appear, there are no attachment files.]

To ensure your figures meet our technical requirements, please review our figure guidelines: https://journals.plos.org/plosone/s/figures

You may also use PLOS’s free figure tool, NAAS, to help you prepare publication quality figures: https://journals.plos.org/plosone/s/figures#loc-tools-for-figure-preparation.

NAAS will assess whether your figures meet our technical requirements by comparing each figure against our figure specifications.

Attachment Submitted filename: report_10.64898+2026.07.02.736004.pdf

# Author comment: Author response to Decision Letter 1

**DOI:** 10.1371/journal.pone.0353900.r002

22 Sep 2026

Response to Reviewers

We sincerely thank the reviewer for the detailed and constructive assessment of our manuscript. We have carefully considered each point raised and have made substantial revisions throughout the manuscript, with individual responses provided below.

We particularly appreciate the reviewer’s recognition that the establishment and immune characterisation of the TAM6 and WIN6 models represents a contribution beyond the specific therapeutic findings reported here. We also agree that dissemination of well-controlled negative preclinical findings is important, particularly for therapeutic strategies that are being actively considered for clinical translation, as is the case for CD47-targeted immunotherapy.

In response to the reviewer’s central concerns, we have strengthened the quantitative reporting of the in vivo studies by adding hazard ratios, 95% confidence intervals and corresponding statistical comparisons for the key efficacy analyses. We have also substantially revised the Discussion to more clearly distinguish conclusions directly supported by the present data from hypotheses arising from those findings. In particular, we now explicitly acknowledge the absence of pharmacokinetic and target-engagement measurements, the lack of on-treatment or post-treatment tumour microenvironment profiling and the limitations of the toxicity assessment.

More broadly, mechanistic interpretations have been revised throughout to use appropriately conditional language, and proposed explanations for the lack of in vivo efficacy are now presented as hypotheses requiring further investigation rather than as established mechanisms.

We believe these revisions have substantially strengthened the manuscript and aligned the conclusions more closely with the evidence presented. We hope that the revised version now addresses the reviewer’s concerns in full.

Comments

#1. The manuscript concludes that ALX301 does not improve efficacy in vivo, but no effect size, hazard ratio, or confidence interval is reported for any of the key in vivo comparisons in Figures 5, 6, or 7. Reporting only that a comparison is not significant is not sufficient to support a negative claim of this kind. Without a quantified effect size and its confidence interval, it is not possible for the reader to distinguish a true absence of effect from an underpowered study that simply failed to detect a real but modest effect. This is especially important given that no a priori power calculation was performed, as the authors themselves acknowledge.

We thank the reviewer for this important suggestion, which has strengthened the quantitative support for the central negative finding of the manuscript. We have now added effect estimates for the key in vivo survival comparisons in Figures 5-7. Hazard ratios with 95% confidence intervals, together with the corresponding log-rank P values, are reported in the revised figure legends.

We have also added the relevant P values directly to the main survival figures in Figures 5-7, specifically for the treatment-group comparisons being evaluated in the manuscript. The same approach has been applied to Supplementary Figure 2, where the anti-GD2 dose-finding experiment is shown.

In Supplementary Figure 2, the medium-dose anti-GD2 group demonstrated a significant survival benefit compared with vehicle, supporting selection of this dose for the subsequent combination studies with ALX301.

For the reviewer’s convenience, these additions have been highlighted within the revised figure legends in the marked-up manuscript on pages 29-30 for Figures 5-7 and page 31 for Supplementary Figure 2.

#2. There is no pharmacokinetic or pharmacodynamic data supporting that ALX301 achieved adequate exposure or target engagement in vivo. No blood or tumor concentration data, half life, or receptor occupancy data for CD47 are provided. The statement that the same reagent lot was used in vitro and in vivo does not address this concern, since lot consistency does not establish that the drug reached the tumor at a concentration sufficient to block CD47 in that tissue. Without this evidence, the negative in vivo result cannot be distinguished from a dosing or exposure failure rather than a true biological lack of efficacy.

We thank the reviewer for this important point and agree that greater emphasis is required in the manuscript to point the reader to the lack of definitively proven failure of therapeutic effect of ALX301 in vivo.

We have therefore revised the fourth paragraph of the Discussion to explicitly acknowledge this limitation. Moreover, we have also clarified later in the same paragraph that activity of the same ALX301 preparation in our in vitro assays demonstrates reagent functionality only and does not establish adequate in vivo exposure or tumour target engagement. We include the following text (pages 20-21 highlighted):

“Pharmacokinetic measurements including circulating or intratumoural ALX301 concentrations and CD47 receptor occupancy were not assessed in the present study. We therefore cannot exclude inadequate tumour exposure or incomplete target engagement as contributors to the absence of detectable therapeutic benefit. The activity of the same ALX301 preparation in our in vitro assays confirms reagent functionality but does not establish in vivo exposure or tumour target engagement.”

#3. The Discussion attributes the lack of in vivo benefit to a myeloid rich, immunosuppressive tumor microenvironment, but this characterization is based exclusively on untreated tumors collected outside the treatment window, as stated in the Methods. No on treatment or post treatment tumor microenvironment data are presented. The manuscript should either tone down this mechanistic claim considerably, using more conditional language throughout, or acknowledge more clearly that this is a hypothesis for future testing rather than a finding supported by the present data.

We thank the reviewer for this important point and agree that the mechanistic interpretation should be more clearly qualified and presented as a hypothesis for future investigation rather than as a conclusion established by the present data.

Accordingly, we have substantially revised the Discussion, as well as related wording throughout the manuscript, to avoid presenting the baseline myeloid-rich tumour microenvironment as a definitive mechanism of resistance to ALX301.

In the fifth paragraph of the Discussion (page 21 line 424), we now describe the tumour microenvironment as a potential explanation for the lack of in vivo efficacy and explicitly acknowledge that the immune profiling was performed on untreated tumours collected outside the therapeutic experiments. We therefore state that the present data do not prove that myeloid composition caused resistance to ALX301.

Later in the same paragraph, we also explicitly acknowledge that no on-treatment or post-treatment tumour microenvironment profiling was performed. The revised Discussion now emphasises that dedicated longitudinal profiling will be required to determine whether treatment alters macrophage state, myeloid composition or antigen-presentation programmes, and whether these features are associated with responsiveness to CD47 blockade.

For the reviewer’s convenience, these revisions have been highlighted in the marked-up manuscript.

#4. Toxicity was assessed using a single complete blood count timepoint at day 19 or at humane endpoint. A single timepoint cannot establish whether the observed reduction in hemoglobin and hematocrit is transient or progressive. A time course including earlier timepoints, for example days 3, 7, and 14, would be needed to properly characterize the safety profile of ALX301 in combination with chemoimmunotherapy, particularly since red cell effects are described as an expected, on target consequence of CD47 blockade that could plausibly recover or worsen with continued dosing.

We thank the reviewer for this important comment and agree that a single blood count timepoint cannot determine whether the observed reductions in haemoglobin and haematocrit were transient, progressive or recovering over time.

We have therefore revised the third paragraph of the discussion (page 20 line 400) to state this explicitly as a limitation. We now clarify that blood was collected only at day 19 or at humane endpoint, that the kinetics of these haematological changes cannot be determined from the present study, and that serial haematological monitoring would be required to more fully characterise the safety profile of ALX301 in combination with chemoimmunotherapy.

We have also moderated the interpretation of the safety data accordingly (page 18 line 366). While no overt clinical or histopathological toxicity was observed under the conditions tested, we no longer present the available blood count data as sufficient to establish the temporal profile of treatment-related haematological effects.

For the reviewer’s convenience, these revisions have been highlighted in the marked-up manuscript.

#5. Several in vivo cohorts are very small, for example n equals 3 for the TAM6 vehicle and combination groups in Figure 5b. Combined with the absence of a power calculation, this raises the concern that the study may simply be underpowered to detect a real effect in this particular model, rather than providing strong evidence that no effect exists. The authors should at minimum discuss the limitations this places on interpretation of the TAM6 findings specifically.

Thank you for highlighting the small group sizes and the resulting limitations in statistical power. In response to this comment, we performed a thorough re-review of the raw in vivo data and identified that several treated animals had previously been excluded from the plotted analyses. On reassessment, we determined that the exclusion criterion applied to these animals was not appropriate. We have therefore revised the analyses to include all treated animals enrolled in these experiments.

This has increased the group sizes in the affected cohorts, and the corresponding figures statistical analyses and underlying source data have been updated accordingly (Figure 5b, legend page 29-30). Importantly, inclusion of these animals did not alter the overall conclusions of the experiments, but provides a more complete representation of the treated cohorts.

The Methods have also been corrected to state explicitly that all treated animals enrolled in the studies are included in the final analysis (page 10 line 316).

#6. Blinding is explicitly described only for humane endpoint assessment and calliper based tumor measurement. It is not stated whether drug administration, complete blood count analysis, or histopathological scoring of heart, lung, kidney, liver, spleen, and tumor sections were performed with the investigator blinded to treatment allocation. Given that the histopathology conclusion of no tissue injury and the hematology comparisons both involve an element of interpretation, blinding status for these assessments should be clarified.

We thank the reviewer for requesting clarification regarding blinding.

Drug administration was not performed blinded to treatment allocation. A practical limitation was that temozolomide and irinotecan were diluted in PBS immediately prior to administration to minimise the risk of precipitation or loss of solubility, meaning that treatment syringes could not be prepared substantially in advance and independently relabelled for blinded dosing. We have clarified this in the Methods under In vivo studies and treatment combinations (highlighted on page 10 line 190-192).

Importantly, tumour measurements and humane endpoint assessments were performed blinded to treatment allocation, thereby reducing the potential for treatment knowledge to influence the primary efficacy assessments (Methods page 12 line 401).

Complete blood count analysis was performed externally by IDEXX UK. Histological assessment was not performed using a formal blinded scoring system. We have clarified both points in the Methods (page 11 line 373).

We have also made clear in the Methods section entitled Blood collection and histology (page 11 line 360) that the histological assessment was qualitative, that no formal histopathological scoring was performed, and that sections were not independently reviewed by a veterinary pathologist. Accordingly, we have moderated the wording in the discussion so that the absence of overt histological abnormalities is presented as supportive evidence of tolerability rather than as definitive evidence of an absence of tissue toxicity (page 20 from line 756).

#7. The selection of ALX301 as the sole CD47 blocking agent tested in vivo is justified in the Discussion primarily on the basis of an anticipated favorable toxicity profile relative to Fc competent anti CD47 antibodies. Since this is the only in vivo agent tested and no in vivo dose escalation beyond the schedule used was performed, the rationale for fixing both the agent and the dose at a single level determined by provider guidance should be stated more explicitly, and the implications of not testing higher exposure should be discussed as a clear limitation rather than mentioned only briefly.

We have now explicitly noted in the results that the complete response rate was numerically lower in the chemoimmunotherapy plus ALX301 group than in the chemoimmunotherapy backbone group (4/7 versus 5/7, respectively), although this was not accompanied by a significant survival difference. This addition has been highlighted in the Results section entitled “Intensified CD47 blockade with chemoimmunotherapy showed favourable safety profile but no additional benefit.” (highlighted on page 18 lines 362-265).

We have also taken this comment into account more broadly when revising the manuscript and have ensured that potentially relevant differences suggesting reduced efficacy with ALX301 are reported and appropriately contextualised. For example, in the WIN6 anti-GD2 monotherapy experiment, the anti-GD2-only group showed a significant survival advantage over the anti-GD2 plus ALX301 group, including one complete response. We now report this finding explicitly, while also noting that it was not reproduced in TAM6 or in subsequent experiments and therefore does not provide consistent evidence that ALX301 antagonises anti-GD2 activity. This addition has been highlighted in the Results section entitled “CD47 blockade does not improve anti-GD2 antibody or chemoimmunotherapy in vivo.” (page 17 line 333).

#9. Several passages in the Discussion use assertive, declarative language, for example stating that the findings are suggesting that the myeloid niche limits benefit, when the underlying evidence is associative rather than mechanistic. The authors should revise this language throughout to more conditional wording such as may or could, so that the strength of the claims matches the strength of the supporting data.

As also addressed in response to Comment #3, we have revised the Discussion extensively throughout to ensure that the strength of the language more appropriately reflects the associative nature of the supporting data.

In particular, proposed explanations relating to the tumour microenvironment are now presented using conditional language and framed as hypotheses for future investigation rather than as mechanisms directly demonstrated by the present study. We have also revised related wording elsewhere in the manuscript to ensure that conclusions remain aligned with the strength of the available evidence.

#10. The Data Availability Statement indicates only that data will be made available upon acceptance, without specifying a repository or identifier. A more specific statement identifying where the minimal dataset will be deposited would strengthen compliance with data sharing expectations.

A full data package has been prepared for upload and will be deposited in Open Science Framework upon manuscript acceptance.

#11. There are several recurring typographical errors that should be corrected throughout, including Institute of Chid Health, which should read Institute of Child Health, and Research into Chidhood Cancer, which should read Research into Childhood Cancer, along with buffer and from, which appear as bufffer and ffrom in the Methods.

Thank you for such careful reading of the manuscript. We have carefully proofread it and corrected the specific errors identified, including “Institute of Child Health”, “Research into Childhood Cancer”, “buffer” and “from”, as well as other typographical inconsistencies identified throughout the text.

#12. The schematic in Figure 1a states that the N297A mutation renders the Fc region of ALX301 inactive. This is not entirely accurate, since N297A abrogates Fc gamma receptor engagement specifically while the CD47 binding function of the SIRP alpha domain is preserved. The figure legend or label should be revised for precision.

We have revised the Figure 1a annotation adjacent to the Fc portion of ALX301 to describe the effect of the N297A mutation more precisely. ALX301 is now described as comprising an engineered SIRPα D1 domain fused to a mouse IgG1 Fc containing the N297A mutation, which abrogates Fcγ receptor engagement while preserving CD47 binding. This replaces the previous, less precise description of the Fc region as “inactive”.

#13. Reference 20 lists author initials only, for example T B, W L, N B, rather than full author names, and should be corrected to a standard citation format.

Thanks for pointing this out. The reference has been corrected to include the appropriate author names and initials rather than initials alone. We have also reviewed the complete reference list and corrected additional inconsistencies.

Attachment Submitted filename: Response to Reviewers.docx

# Peer review: Decision Letter 1

**DOI:** 10.1371/journal.pone.0353900.r003

**Authors:** Lu Zhang

1 Oct 2026

CD47 blockade augments anti-GD2 driven phagocytosis in vitro but fails to improve in vivo efficacy in immunocompetent, chemoresistant neuroblastoma preclinical models

PONE-D-26-32803R1

Dear Dr. Courtney Himsworth,

We’re pleased to inform you that your manuscript has been judged scientifically suitable for publication and will be formally accepted for publication once it meets all outstanding technical requirements.

Within one week, you’ll receive an e-mail detailing the required amendments. When these have been addressed, you’ll receive a formal acceptance letter and your manuscript will be scheduled for publication.

An invoice will be generated when your article is formally accepted. Please note, if your institution has a publishing partnership with PLOS and your article meets the relevant criteria, all or part of your publication costs will be covered. Please make sure your user information is up-to-date by logging into Editorial Manager at Editorial Manager® and clicking the ‘Update My Information' link at the top of the page. For questions related to billing, please contact billing support.

If your institution or institutions have a press office, please notify them about your upcoming paper to help maximize its impact. If they’ll be preparing press materials, please inform our press team as soon as possible -- no later than 48 hours after receiving the formal acceptance. Your manuscript will remain under strict press embargo until 2 pm Eastern Time on the date of publication. For more information, please contact onepress@plos.org.

Kind regards,

Lu Zhang

Academic Editor

PLOS One

Additional Editor Comments (optional):

Reviewers' comments:

Reviewer's Responses to Questions

**Comments to the Author**

Reviewer #3: All comments have been addressed

**********

2. Is the manuscript technically sound, and do the data support the conclusions?

Reviewer #3: Yes

**********

3. Has the statistical analysis been performed appropriately and rigorously?

Reviewer #3: Yes

**********

4. Have the authors made all data underlying the findings in their manuscript fully available?

The PLOS Data policy

Reviewer #3: Yes

**********

5. Is the manuscript presented in an intelligible fashion and written in standard English?

Reviewer #3: Yes

**********

Reviewer #3: This study revealed the constraint of CD47 in macrophage cancer therapy. Although CD47 is promoted as don't eat me signal and considered as a key regulator of phagocytosis, its significance in macrophage immunotherapy in vivo is not clear. This study demonstrated that CD47 targeting does not contribute to macrophage cancer therapy in vivo, which is a key difference between in vitro observations.

The revised MS addressed the reviewer's questions well. The addition of stat power reinforces their conclusion that CD47 targeting does not improve macrophage cancer therapy in vivo.

**********

what does this mean?). If published, this will include your full peer review and any attached files.

If you choose “no”, your identity will remain anonymous but your review may still be made public.

**Do you want your identity to be public for this peer review?**  For information about this choice, including consent withdrawal, please see our Privacy Policy

Reviewer #3: No

**********

# Editor report: Acceptance letter

**DOI:** 10.1371/journal.pone.0353900.r004

**Authors:** Lu Zhang

PONE-D-26-32803R1

PLOS One

Dear Dr. Himsworth,

I'm pleased to inform you that your manuscript has been deemed suitable for publication in PLOS One. Congratulations! Your manuscript is now being handed over to our production team.

At this stage, our production department will prepare your paper for publication. This includes ensuring the following:

* All references, tables, and figures are properly cited

* All relevant supporting information is included in the manuscript submission,

* There are no issues that prevent the paper from being properly typeset

You will receive further instructions from the production team, including instructions on how to review your proof when it is ready. Please keep in mind that we are working through a large volume of accepted articles, so please give us a few days to review your paper and let you know the next and final steps.

Lastly, if your institution or institutions have a press office, please let them know about your upcoming paper now to help maximize its impact. If they'll be preparing press materials, please inform our press team within the next 48 hours. Your manuscript will remain under strict press embargo until 2 pm Eastern Time on the date of publication. For more information, please contact onepress@plos.org.

You will receive an invoice from PLOS for your publication fee after your manuscript has reached the completed accept phase. If you receive an email requesting payment before acceptance or for any other service, this may be a phishing scheme. Learn how to identify phishing emails and protect your accounts at https://explore.plos.org/phishing.

If we can help with anything else, please email us at customercare@plos.org.

Thank you for submitting your work to PLOS One and supporting open access.

Kind regards,

PLOS One Editorial Office Staff

on behalf of

Dr. Lu Zhang

Academic Editor

PLOS One
